# A novel identified epithelial ligand-receptor-associated gene signature highlights POPDC3 as a potential therapy target for non-small cell lung cancer

**DOI:** 10.1038/s41419-025-07410-9

**Published:** 2025-02-19

**Authors:** Xiao-Ren Zhu, Jia-Qi Zhu, Qian-Hui Gu, Na Liu, Jing-Jing Lu, Xiao-Hong Li, Yuan-Yuan Liu, Xian Zheng, Min-Bin Chen, Yong Ji

**Affiliations:** 1 Department of Radiotherapy and Oncology, Affiliated Kunshan Hospital of Jiangsu University, Kunshan, China; 2 Clinical Research and Lab Center, Affiliated Kunshan Hospital of Jiangsu University, Kunshan, China; 3 Medical School of Jiangsu University, Zhenjiang, China; 4 Department of Hepatobiliary and Pancreatic Surgery, Affiliated Hospital of Nantong University, Medical School of Nantong University, Nantong, China; 5 Department of Clinical Laboratory, The First People’s Hospital of Taicang, Taicang, China; 6 Department of Pharmacy, Affiliated Kunshan Hospital of Jiangsu University, Kunshan, China; 7 Department of Thoracic Surgery, The Affiliated Wuxi People’s Hospital of Nanjing Medical University, Wuxi People’s Hospital, Wuxi Medical Center, Nanjing Medical University, Wuxi, China

**Keywords:** Non-small-cell lung cancer, Oncogenes

## Abstract

The tumor microenvironment (TME) is pivotal in non-small cell lung cancer (NSCLC) progression, influencing drug resistance and immune cell behavior through complex ligand-receptor (LR) interactions. This study developed an epithelial LR-related prognostic risk score (LRrisk) to identify biomarkers and targets in NSCLC. We identified twenty epithelial LRs with significant prognostic implications and delineated three molecular NSCLC subtypes with distinct outcomes, pathological characteristics, biological pathways, and immune profiles. The LRrisk model was constructed using twelve differentially expressed ligand-receptor interaction-related genes (LRGs), with a focus on POPDC3 (popeye domain-containing protein 3), which was overexpressed in NSCLC cells. Functional assays revealed that POPDC3 knockdown reduced cell proliferation, migration, invasion, and epithelial-mesenchymal transition (EMT), while its overexpression promoted cancerous activities. In vivo, POPDC3 silencing hindered, and its overexpression accelerated the growth of NSCLC xenografts in nude mice. Additionally, high expression levels of POPDC3 in NSCLC tissues were associated with enhanced CD4^+^ T cell infiltration and increased PD-1 expression within the TME. Moreover, ectopic POPDC3 overexpression in C57BL/6 J mouse Lewis lung carcinoma (LLC) xenografts enhanced CD4^+^ T cell infiltration and PD-1 expression in the TME. This research establishes a robust epithelial LR-related signature, highlighting POPDC3 as a critical facilitator of NSCLC progression and a potential therapeutic target.

## Introduction

Lung cancer remains a dominant cause of cancer-induced mortality globally [1], with NSCLC accounting for approximately 85% of all cases [2]. A significant proportion of NSCLC patients are diagnosed with metastatic and other advanced stages at the onset [3]. While advancements in surgery, immunotherapy, chemotherapy, and targeted approaches have improved survival for numerous NSCLC patients [4–7], the 5-year survival remains disappointingly low for advanced patients, underscoring the necessity for new predictive biomarkers beyond traditional clinical staging and histopathological assessments [8].

The tumor microenvironment (TME) is a critical factor in tumor progression and metastasis [9]. Single-cell RNA sequencing (scRNA-seq) enables detailed characterization of the cellular composition and transcriptional states of different TME cells, including epithelial cells, fibroblasts, macrophages, T cells, and B cells [10]. Epithelial cells, in particular, are central to TME dynamics, secreting a range of ligands such as growth factors, cytokines, and chemokines [11, 12]. These ligands bind to specific receptors on the surface of immune and stromal cells, modulating their behavior, thus fostering an environment conducive to tumor cell survival, proliferation, metastasis, and progression [13, 14]. Consequently, exploring ligand-receptor (LR) interactions between epithelial cells and other TME constituents is crucial for precise patient risk stratification and the development of effective NSCLC therapies [15]. Such interactions have been shown to affect disease subtypes and prognostic outcomes in various cancers, including triple-negative breast cancer [16], melanoma [17], colon adenocarcinoma [18], and bladder cancer [19]. Despite the significant impact of multi-omics profiling in revealing the biological heterogeneity of NSCLC, there remains a scarcity of studies focusing on LR interactions within NSCLC-associated epithelial cells. Further exploration of their relationship with molecular subtypes, clinical features, and prognostic significance is therefore essential.

This study introduces an epithelial LR interaction-based gene prognostic risk score (LRrisk) for NSCLC using TCGA (The Cancer Genome Atlas) dataset. The association of LRrisk with immune signatures and biomarkers predictive of immunotherapy response was examined. Significantly, the bioinformatic results were also confirmed by analyzing human NSCLC tissue samples, cultivating NSCLC cells in vitro, and using subcutaneous xenograft models. Our findings provide new insights into the interaction between LR dynamics and NSCLC, highlighting the significance of popeye domain-containing protein 3 (POPDC3) in promoting immune evasion and tumor progression.

## Materials and methods

### Reagents and chemicals

DAPI (4′,6-diamidino-2-phenylindole) and EdU (5-ethynyl-2’-deoxyuridine) were obtained from Thermo-Fisher Invitrogen (Shanghai, China). Puromycin and culture medium were obtained from Sigma-Aldrich (St. Louis, MO). The primary antibodies used were the following: anti-POPDC3 (1:2000; 11800-1-AP; ProteinTech), anti-anillin (ANLN, 1:2000; A03997-1; BOSTER), anti-Desmoglein 2 (DSG2, 1:2000; BA3606; BOSTER), anti-CUB domain containing protein 1 (CDCP1, 1:2000; PB0978; BOSTER), anti-stanniocalcin 2 (STC2, 1:2000; 60063-1-lg; proteintech), anti-RHOV (1:500; sc-515072; Santa Cruz Biotechnology), anti-E-cadherin (1:2000; BM4166; BOSTER), anti-N-cadherin (1:2000; BA0673; BOSTER), anti-Vimentin (1:2000; BM4029; BOSTER), anti-SNAI1 (1:2000; A00716-2; BOSTER), anti-Slug (1:2000; PB9439; BOSTER), anti-Bcl2 (1:2000; A00040-1; BOSTER), anti-Bax (1:2000; A00183; BOSTER), anti-Ki67 (1:4000; ab16667; Abcam), anti-GAPDH (1:4000; ab8245; Abcam), anti-CD4 (1:1600; ab237722; Abcam), anti-PD-1 (1:1000; 18106-1-AP; ProteinTech).

### Data acquisition

scRNA-seq data (GSE117570) for NSCLC and associated bulk-tissue RNA-sequencing datasets (GSE31210, GSE50081, and GSE3134) were retrieved from the Gene Expression Omnibus (GEO) database. The scRNA-seq dataset encompassed eight samples from four patients, comprising four NSCLC tumor samples and four adjacent normal tissue samples. Additionally, RNA-sequencing (RNA-seq) data along with clinical information for 994 NSCLC patients were sourced from the TCGA database to form the training cohort. Validation was performed using three additional GEO datasets.

### Data analysis for scRNA-seq

Analysis was conducted using R (version 4.1.3) and the Seurat R package (version 4.2.0). The Seurat R package was utilized to create objects for each sample by processing the cell-by-gene count matrices using the CreateSeuratObject function, with a minimum presence criterion of three cells per gene. Cells were filtered out if they had a significant proportion of mitochondrial or erythrocyte genes greater than 15% or if the gene count was uncharacteristically low (below 100) or high (above 7000), suggesting suboptimal cell integrity. After quality control, a total of 10,664 cells were retained for further analysis. Data normalization and scaling were performed using NormalizeData and ScaleData functions, respectively. Highly variable genes were identified using the FindVariableFeatures function and principal component analysis (PCA) was applied. Significant principal components selected using the JackStraw and ElbowPlot functions were utilized for t-distributed stochastic neighbor embedding (t-SNE) and Uniform Manifold Approximation and Projection (UMAP) visualization. Clustering was performed using Seurat’s FindClusters function set at a resolution of 0.6. Differentially expressed genes between tumor and normal cell clusters, were identified using the FindMarkers function, with a significance threshold set at a *p*-value of less than 0.05, and a log fold change of more than 1.

### Cell–cell communication analysis

CellPhoneDB was utilized to investigate cellular interactions [20]. For this analysis, gene expression matrices derived from the scRNA-seq datasets were input into CellPhoneDB, along with a list of established ligands and receptors (LRs). This tool then assessed the prevalence of ligand and receptor (LR) interactions among various cell types and produced a compilation of potential cell–cell communication networks.

### Development of LR subtypes using consensus clustering

The “ConsensusClusterPlus” tool was employed to define clusters based on the expression patterns of prognostically relevant LR in NSCLC. The clustering heatmap was generated using the “pheatmap” package in R. The optimal cluster count was generated by examining the consensus cumulative distribution function (CDF) plot and the delta area plot, with the criteria being high intra-cluster consistency, low coefficient of variation, and a plateau in the area under the CDF curve, indicating no significant increase.

### Functional enrichment analysis

This analysis involved retrieving Hallmark Gene sets from the Molecular Signatures Database (MSigDB) to examine gene functions. Gene Set Enrichment Analysis (GSEA) was carried out on the LR clusters using the GSEA software, focusing on pathways that exhibited significant enrichment as indicated by normalized enrichment scores (NES). Pathways with a false discovery rate (FDR) of less than 0.05 were deemed statistically significant.

### Development of the LRrisk

LASSO is utilized as both a regularization and dimension reduction method, particularly useful in biomarker screening for survival analysis when combined with the Cox model [21]. Differentially expressed genes (DEGs) between the C1 and C3 clusters were identified using the DESeq2 package in R, specifically those with a false discovery rate (FDR) below 0.05. The top 500 DEGs were then analyzed using univariate Cox analysis within the TCGA dataset, identifying 94 genes associated with disease progression (*P* < 0.05). To further refine the candidate genes, lasso logistic regression with 10-fold cross-validation was applied using the “glmnet” package in R. This was followed by the application of a multivariate Cox proportional hazards model that included stepwise regression for subsequent gene selection. The risk score formula was defined as Risk. Score =$\sum (\beta i\times Exp{i})$, where Exp*i* represents the expression level of each gene related to intercellular communication and *β* is the coefficient derived from the multivariate Cox regression analysis. Risk scores were calculated for each participant using a cutoff of 0.0355 to classify them into high- and low-risk groups. The predictive accuracy of the LRrisk score for 1-year, 2-year, and 3-year survival rates was evaluated through ROC analysis, employing the “survivalROC” package in R to determine the sensitivity and specificity, and AUC. This approach was validated in the GSE31210, GSE50081, and GSE3134 datasets to ensure the generalizability of the LRrisk model.

### Analysis of tumor immune signatures and function enrichment

The “ESTIMATE” R package was used to derive immune and stromal scores from gene expression data, estimating the proportion of the extracellular matrix and immune cells within tumor specimens. Higher scores indicated a more substantial presence within the tumor microenvironment (TME). The presence of 22 types of immune cells in NSCLC tissues was quantified using the CIBERSORT algorithm [22]. Meanwhile, the association between the LR score and the expression levels of immune checkpoint genes was analyzed using the Wilcoxon test, with the findings presented in box plots. Additionally, the Tumor Immune Dysfunction and Exclusion (TIDE) framework was utilized to predict patient responses to immune checkpoint blockade (ICB) therapy by modeling the gene signatures indicative of two immune evasion mechanisms [23].

### Clinical samples and Immunohistochemical (IHC) analysis

Two tissue microarrays acquired from Shanghai Outdo Biotech Co., Ltd., (Shanghai, China) were utilized in this study. These arrays contained a total of 360 matched pairs of NSCLC and adjacent non-tumorous lung tissues. Selection and categorization of the paired samples were rigorously performed based on clinical data. The study consisted of 180 NSCLC patients, equally divided between lung adenocarcinoma and squamous cell carcinoma, with a demographic breakdown of 135 males and 45 females. The patients’ age ranged from 31 to 80 years, with an average age of 61.2 years. For IHC analysis, tissue sections underwent antigen retrieval and blocking, followed by overnight incubation with anti-POPDC3 antibody (ProteinTech) at 4 °C. After washing with PBS, sections were treated with a secondary antibody for one hour and developed with diaminobenzidine (DAB) (Servicebio). Subsequently, hematoxylin was used for counterstaining, and the sections were mounted with neutral resin for examination under a fluorescence microscope (Nikon). Two independent pathologists evaluated the IHC staining, achieving consensus on the scoring.

### Multiplex immunofluorescence immunohistochemistry and imaging

The collection of tissue samples was conducted in compliance with ethical guidelines, ensuring that informed consent was secured from each participant. The tissue microarray was prepared for multiplex immunofluorescence analysis following a protocol established by previous research [24]. In summary, the process involved baking the microarray at 63 °C for 1 h, followed by deparaffinization with an automated stainer (LEICAST5020, LEICA). Antigen retrieval was then performed, and endogenous peroxidase activity was quenched using a commercial hydrogen peroxide solution for 10 min. The microarray was blocked for 10 min and subsequently incubated with specific primary antibodies for immunofluorescence—POPDC3, PD1, CD4, CD8, and PAN-CK—each at optimal dilutions, for one hour. After a wash with TBST, a secondary antibody was applied for 10 min, followed by an incubation with Opal dye. Between each indicator, microwave treatment was used to strip the antibody complex, and this cycle was repeated as necessary. The final step involved nuclear counterstaining with DAPI for five minutes before the slides were preserved with an antifade mounting medium.

### Cell culture and transfection

Established NSCLC cell lines (A549, H1299, H520, H1703, and murine Lewis lung carcinoma/LLC) as well as alveolar epithelial cells (HPAEpic) were purchased from the Cell Bank at the Shanghai Institute of Biological Science (Shanghai, China). HPAEpic and LLC cells were cultured at 37 °C in a humidified atmosphere containing 5% CO_2_, using DMEM supplemented with 10% fetal bovine serum (FBS). The A549 and H1299 cell lines were maintained in the F12K medium with 10% FBS, while H520 and H1703 cells were grown in RPMI1640 also with 10% FBS. Cells were seeded in six-well plates and cultivated overnight. The detailed protocols for obtaining and culturing primary human NSCLC cells (“pri-1#”) were reported previously [25]. The POPDC3-specific shRNA lentivirus (two different sequences, namely “POPDC3-sh-S1/S2”) and the scramble control shRNA (“shC”), lentiviral vectors expressing POPDC3 (“OE-POPDC3”, either murine or human) and the empty vector (“Vec”), were obtained from Genechem (Shanghai, China). Cells were transfected with that lentivirus for 24 h, after puromycin selection at a dosage of 3 µg/mL for 5 days, stable cell lines were established, and verified by qRT-PCR and Western blotting.

### Cellular functional studies and gene/protein detection

qRT-PCR, Western blotting, CCK-8, cell colony formation, EdU staining, phagokinetic track motility, “Transwell,” and “Matrigel Transwell” assays were described in detail in our previous studies [26–28]. The validated mRNA primers were provided by Jian Bio (Suzhou, China). The uncropped blotting images are listed in Fig S5.

### Xenograft model

Both female BALB/c nude mice and female C57BL/6 J mice, 5–6 weeks old, were obtained from the Animal Center of Jiangsu University. Each BALB/c nude mouse received a subcutaneous flank injection with either sh-POPDC3-S1/S2 H1299 cells or control shC H1299 cells (three million cells per mouse) suspended in a 0.20 mL mixture of F12K medium plus Matrigel. Tumor growth was monitored, and tumor size was measured every 5 days. All procedures involving animals were conducted with the approval of the Institutional Animal Care and Use Committee (IACUC) and the Ethics Committee of Jiangsu University. The methodologies for IHC have been described in our previous publications [29]. Alternatively, the nude mice were subcutaneously injected with either OE-POPDC3 priNSCLC-1 cells or control Vec priNSCLC-1 cells (ten million cells per mouse), with priNSCLC-1 xenograft sizes being evaluated after 30 days. The murine LLC cells (three million cells per mouse) with or without POPDC3 OE were injected subcutaneously into the flank area of randomly selected C57BL/6 J mice. After 30 days, the mice were sacrificed and the NSCLC tumor tissues were fixed with 10% formaldehyde for subsequent immunohistochemical staining.

### Statistical analysis

Bioinformatic analysis was carried out using R software, version 4.1.3. Replicates of in vitro experiments were conducted three times, and results were expressed as the mean ± standard deviation (SD). Data normality was verified, enabling the application of suitable statistical methods. Statistical analysis involved one-way ANOVA with post-hoc Scheffe’s test, conducted using software SPSS23.0 (SPSS Inc., Chicago, IL), and this was complemented by Student’s *t*-test where necessary. A *P* value of less than 0.05 was set as the threshold for statistical significance.

## Results

### Identification of ligand-receptor interactions associated with epithelial cells in NSCLC tumor microenvironment

**Fig. 1:**
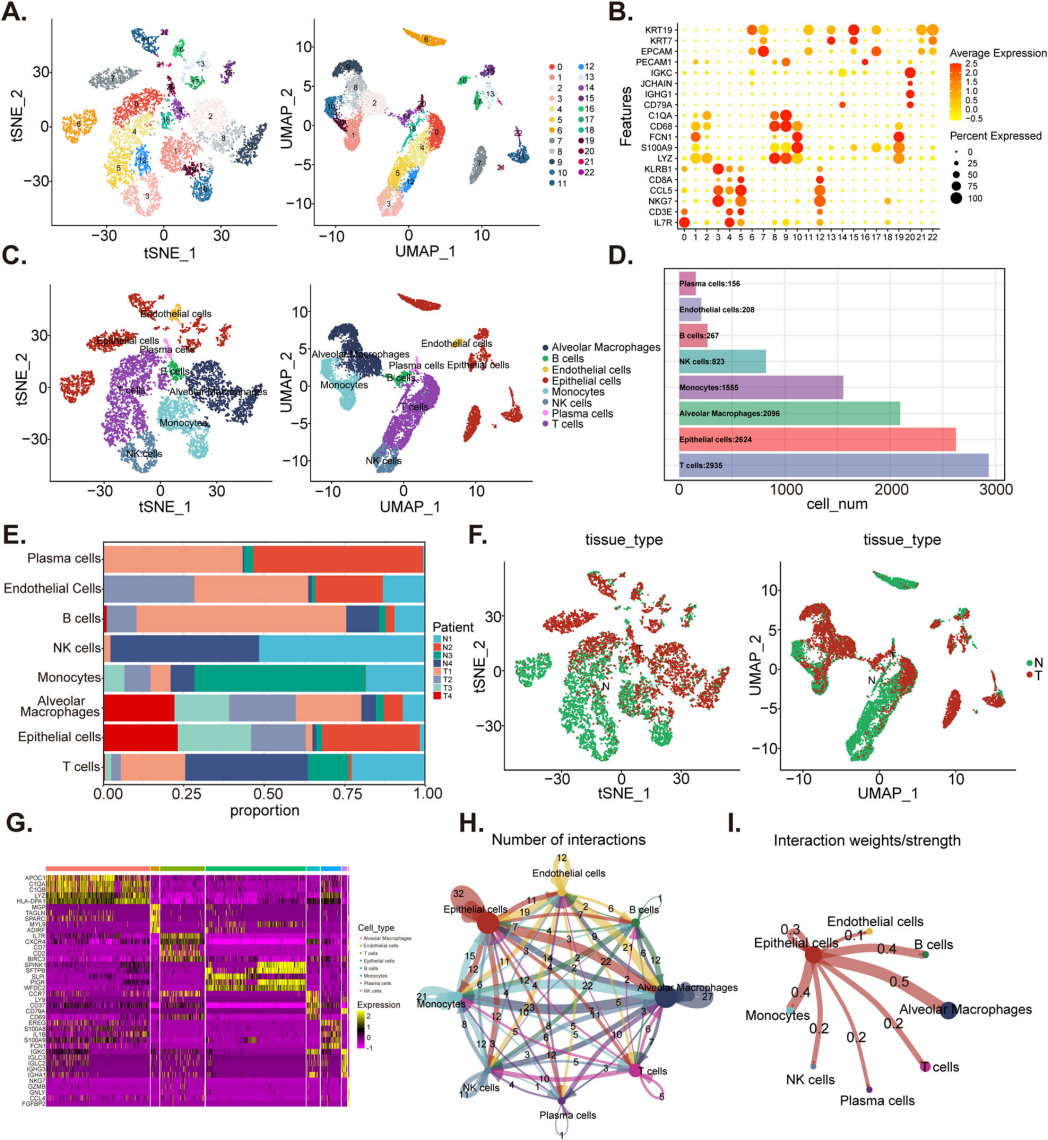
Identification of ligand-receptor interactions associated with epithelial cells in NSCLC tumor microenvironment. tSNE (t-distributed Stochastic Neighbor Embedding) and UMAP (Uniform Manifold Approximation and Projection) of 10,664 cells of four NSCLC samples and their adjacent normal samples (**A**). Dot plot showing the expression pattern of fibroblasts, alveolar macrophages, B cells, epithelial cells and so on (**B**). tSNE and UMAP demonstrated different cell types in these samples (**C**). The number of each cell type in eight samples (**D**). Frequencies of the 8 cell types across the samples are depicted in stacked bar charts (**E**). tSNE and UMAP showing cell distribution in tumor and paracancer samples (**F**). A heatmap reveals the five most prominent marker genes defining various clusters in the NSCLC specimens (**G**). Overview of selected statistically significant interactions between epithelial cells, fibroblasts and others (**H**). Detailed network of cell-cell interaction weights among malignant epithelial cells with other cell subsets (**I**).

Analyzing NSCLC scRNA-seq data from dataset GSE117570 of the GEO database showed no correlation between UMI counts and mitochondrial gene expression, but a strong correlation with mRNA levels (Fig. S1A). Further examination led to the exclusion of cells not meeting quality standards (Fig. S1B, C). The focus then shifted to the top 2000 highly variable genes (Fig. S1D). Despite using PCA to identify 10 principal components, this method was insufficient for clearly segregating different cell types within the tumor-derived cell population (Fig. S1E, F). Consequently, we employed t-SNE (t-distributed stochastic neighbor embedding) and UMAP (Uniform Manifold Approximation and Projection) algorithms, which successfully classified the cells into 23 distinct clusters (Fig. 1A). These clusters were annotated using biomarkers referenced in CellMarker 2.0 [30] and relevant literature [31] (Fig. 1B). The annotated cellular subsets, defined by their expression patterns of marker genes, include T cells (cluster 0, 4, 5, 12, and 18, containing 2935 cells), alveolar macrophages (cluster 2, 8, and 9, containing 2096 cells), B cells (cluster 14, containing 267 cells), monocytes (cluster 1, 10, and 19, containing 1555 cells), NK cells (cluster 3, containing 823 cells), epithelial cells (cluster 6, 7, 11, 13, 15, 17, 21, and 22, containing 2624 cells), endothelial cells (cluster 16, containing 208 cells), and plasma cells (cluster 20, containing 156 cells) (Fig. 1C–E). We illustrated the distribution of tumor and normal tissues and selected tumor tissues for in-depth study (Fig. 1F). Differential gene expression within the TME was assessed using Wilcoxon rank-sum testing, and a heatmap was generated to highlight the top five differentially expressed genes (DEGs) in each cluster (Fig. 1G). CellPhoneDB analysis sheds light on the intricate intercellular communication within NSCLC, uncovering extensive interactions among diverse cell subsets, particularly between epithelial cells and other cell types (Fig. 1H, I). Subsequently, 82 epithelial-related LRs genes were screened for further study (Fig. 1H, I).

### Construction of molecular subtypes of NSCLC based on malignant epithelial ligand-receptor interactions

**Fig. 2:**
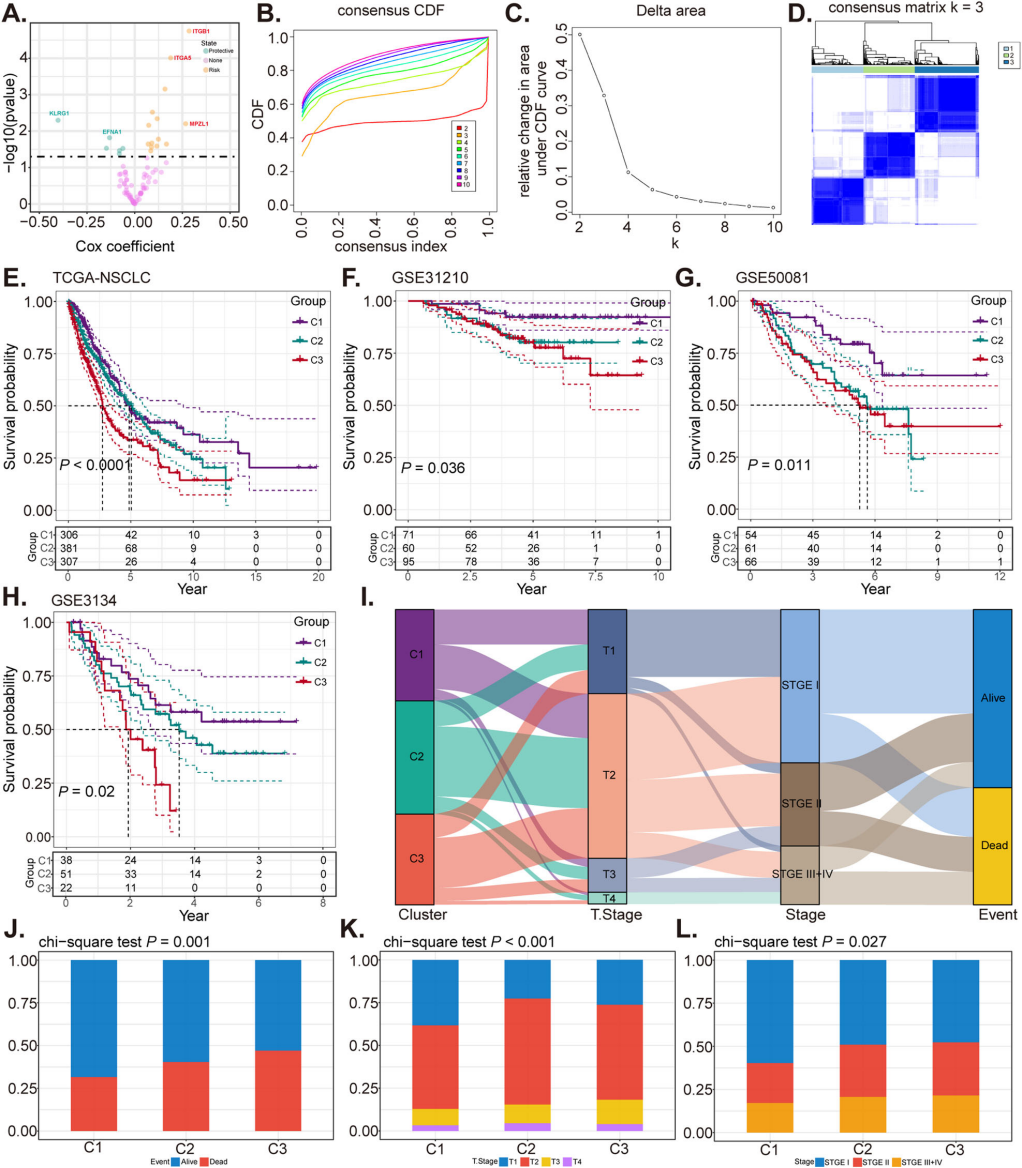
Construction of molecular subtypes of NSCLC based on malignant epithelial ligand-receptor interactions. Volcano map of differential ligand or receptor using univariate Cox analysis (**A**). CDF (Cumulative Distribution Function) curves for *k* = 2–10 in the TCGA-NSCLC cohort (**B**). CDF delta area curve in the TCGA-NSCLC cohort (**C**). Heatmap clustering of TCGA-NSCLC datasets when consensus (*k*) = 3 (**D**). Survival disparities across the molecular subtypes within the TCGA-NSCLC cohort (**E**), as well as in external validation cohorts GSE31210 (**F**), GSE50081 (**G**), and GSE31334 (**H**). The Sankey showed the distribution ratio of 991 samples concerning clinical staging (**I**). Composition percentage of the three clusters in T stage (**J**) and clinical stage (**K**), OS event (**L**).

To further elucidate the significance of epithelial-related LR genes within Bulk RNA-seq datasets, we conducted an investigation focusing on a set of 82 LR genes linked to epithelial characteristics. Among these, 20 LR genes displayed a prognostic association (*P* < 0.05) (Fig. 2A). The optimal cluster count was identified as three (*k* = 3), based on analysis of the cumulative distribution function and the delta area curve (Fig. 2B–D). Notably, the C1 subtype was associated with better outcomes, while the C3 subtype was linked with less favorable prognostic implications (Fig. 2E). Consistently, Kaplan–Meier survival curves revealed shorter survival times in the C3 subtype compared to the C1 subtype in the GSE31210 (*P* = 0.036), GSE50081 (*P* = 0.011), and GSE3134 (*P* = 0.02) datasets (Fig. 2F–H), maintaining consistency with the training set. The distribution ratio of 991 samples concerning clinical staging is illustrated in Fig. 2I. Our research uncovered that patients classified under the C3 subtype, typically associated with a less favorable prognosis, exhibited elevated T stages and overall higher clinical stages, particularly when compared to the C1 subtype. This variation was statistically significant (*P* < 0.05) (Fig. 2J, K). Moreover, the mortality rate was significantly higher in the C3 subtype compared to those classified as C1 or C2 (*P* < 0.05) (Fig. 2L).

### Immune characteristics of different molecular subtypes in NSCLC based on malignant epithelial ligand-receptor interactions

**Fig. 3:**
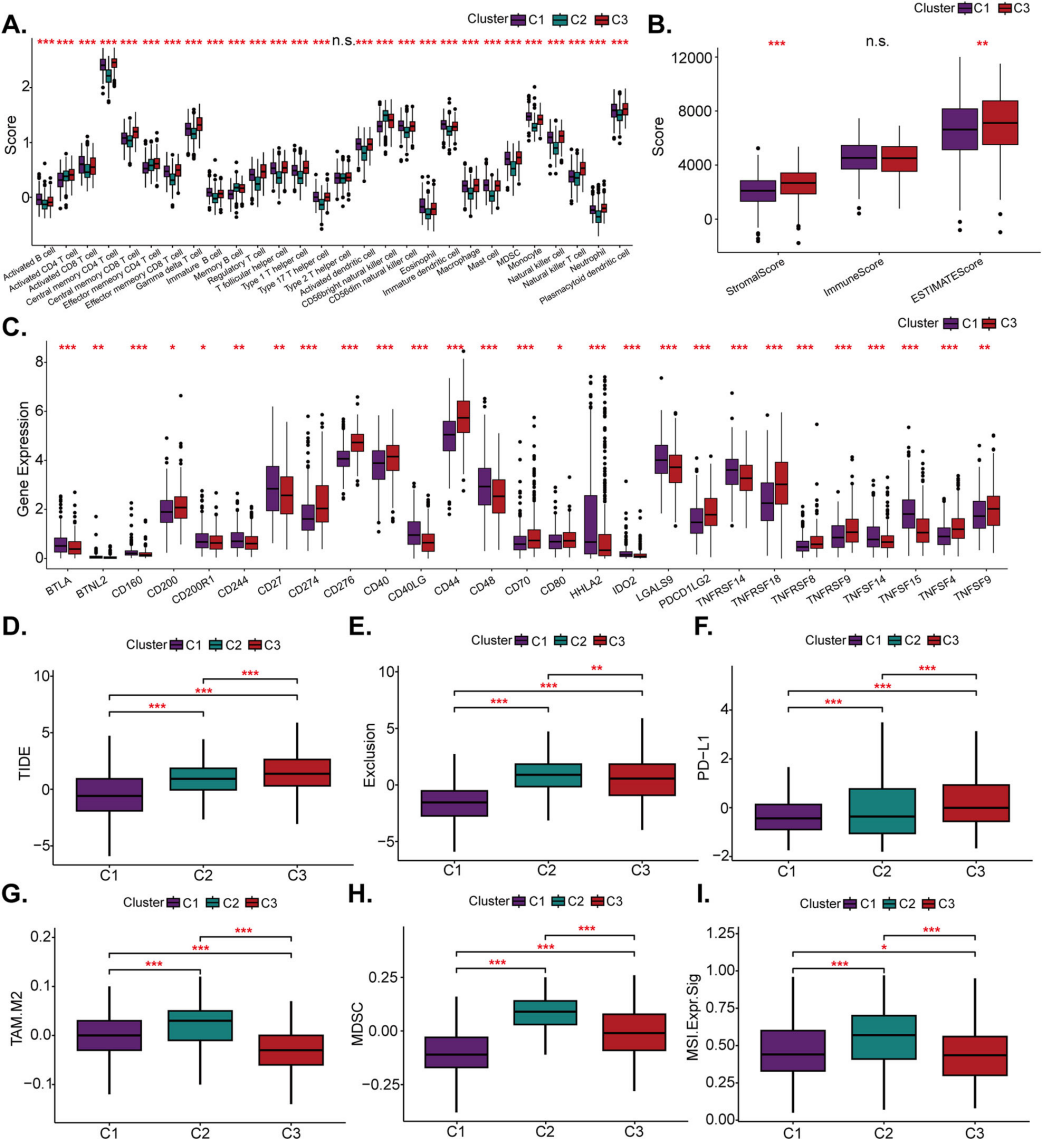
Immune characteristics of different molecular subtypes in NSCLC based on malignant epithelial ligand-receptor interactions. The immune cell scores among the different groups were analyzed using the CIBERSORT algorithm (**A**). Immune cell scoring across groups was also determined through the application of the ESTIMATE algorithm (**B**). Differential expression of immune checkpoint genes between the C1 and C3 subtypes was meticulously examined (**C**). Differences in the TIDE analysis results among the groups were identified, including variations in TIDE scores (**D**), Exclusion scores (**E**), expression of PD-L1 (**F**), levels of TAM.M2 (**G**), prevalence of MDSC (**H**), and expression of MSI (**I**). *P < 0.05; **P < 0.01; ***P < 0.001; “n.s.” stands for *P* > 0.05.

To comprehensively evaluate the variations in the immune microenvironment variations among different molecular subtypes, we quantified the infiltration levels of 28 immune cell types in the TCGA-NSCLC cohort using the single-sample Gene Set Enrichment Analysis (ssGSEA) algorithm. Notably, marked differences in immune cell representation across the molecular subtypes were observed (Fig. 3A). These findings were further supported by the ESTIMATE (Estimation of STromal and Immune cells in MAlignant Tumor tissues using Expression data) algorithm [32, 33], which showed that the C3 subtype of the TCGA cohort had higher ‘StromalScore’ and ‘ESTIMATEScore’ compared to the C1 subtype, suggesting more extensive immune cell infiltration (Fig. 3B). Analysis of immune checkpoint gene expression revealed that the C3 subtype exhibited higher expression of these genes when compared to the C1 subtype (Fig. 3C). It is well-documented that higher TIDE (tumor immune dysfunction and exclusion) scores [23, 34] correlate with lower survival rates and reduced efficacy of immune checkpoint blockade therapies [35]. Our results showed that the C3 subtype was associated with higher TIDE scores, exclusion scores, and increased PD-L1 expression (Fig. 3D–F). Interestingly, the C2 subtype was characterized by an increase in the M2 subtype of tumor-associated macrophages (TAM.M2), myeloid-derived suppressor cells (MDSC), and microsatellite instability (MSI), underscoring the intricate interplay between immune infiltration and patient outcomes in NSCLC (Fig. 3G–I).

### Construction and validation of malignant epithelial ligand-receptor interactions risk (LRrisk) signature in NSCLC

**Fig. 4:**
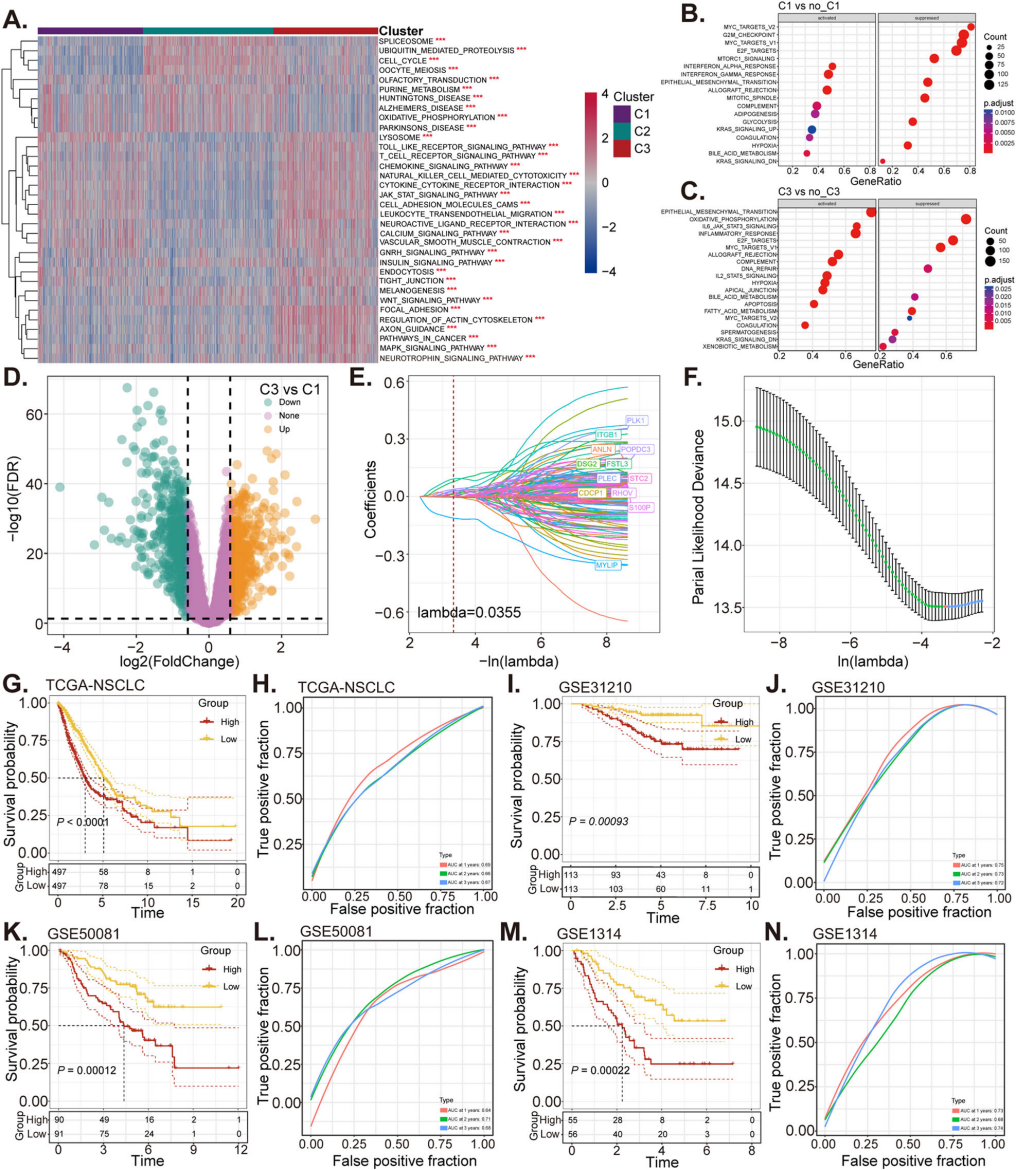
Construction and validation of malignant epithelial ligand-receptor interactions risk (LRrisk) signature in NSCLC. Heat map of enrichment scores of KEGG-related pathways in the three subtypes (**A**). Bubble map of associated pathways in which cluster1 is activated/suppressed (cluster1 vs. no_cluster1 subtype) (**B**). Bubble map of associated pathways in which cluster3 is activated/suppressed (cluster3 vs. no_cluster3 subtype) (**C**). Volcano map of DEGs between C1 and C3 subtype using univariate Cox analysis (**D**). The LASSO regression model is constructed using 12 genes predictive of patient outcomes in the TCGA-NSCLC dataset (**E**, **F**). The KM analysis of the OS between two different groups in TCGA-NSCLC cohort (**G**), GSE31210 (**I**), GSE50081 (**K**) and GSE3134 (**M**) data cohort. The ROC curves were employed to evaluate the model’s predictive accuracy in the TCGA-NSCLC cohort (**H**) as well as in the GSE31210 (**J**), GSE50081 (**L**), and GSE3134 (**N**) cohorts. ***P < 0.001.

The gene set variation analysis (GSVA) package in R is a programming language tailored for statistical analysis, analyzing changes in gene groups across different samples within an experiment without requiring specific labels [36]. This functionality is particularly valuable for examining gene behavior under various conditions [36]. The GSVA package was used to calculate the pathway scores for samples across various molecular subtypes. Notably, we observed significant enrichment of multiple oncogenic pathways, such as JAK-STAT (Janus kinase-signal transducer and activator of transcription), WNT (Wingless/Integrated), and MAPK (mitogen-activated protein kinase), particularly within the C3 subtype (Fig. 4A). Subsequently, employing hall.v7.5.symbols.gmt as a reference for GSEA analysis, we found that the C1 subtype exhibited enhanced activity in the interferon-α response and interferon-γ response pathways, along with reduced activity in the MYC targets and G2-M checkpoint pathways in comparison to the non-C1 subtypes (Fig. 4B). In contrast, the C3 subtype, when compared to the C1/C2 subtypes, showed hyper-activation of pathways such as IL6-JAK-STAT3 signaling and IL2-STAT5 signaling, with the DNA repair and fatty acid metabolism pathways suppressed (Fig. 4C).

To elucidate the differences between the C1 and C3 subtypes, we conducted a differential gene expression analysis, identifying 1137 DEGs (Fig. 4D). Univariate Cox regression analysis of these DEGs revealed 379 genes with prognostic relevance. Further refinement using LASSO-Cox regression analysis selected twelve ligand-receptor interaction-related genes (LRGs, including *PLK1*, *ITGB1*, *ANLN*, *POPDC3*, *DSG2*, *FSTL3*, *PLEC*, *STC2*, *CDCP1*, *RHOV*, *S100P*, and *MYLIP*) for constructing the optimal prognostic signature (Fig. 4E, F). These 12 genes were utilized to develop a risk scoring model (LRrisk), which quantitatively assessed the risk in NSCLC patients. Patients were categorized into low (*n* = 497) and high (*n* = 497) LRrisk groups, with the latter demonstrating reduced survival times (Fig. 4G). The role of AUC (area under the curve) in cancer diagnostics is significant as it helps evaluate how accurately a test, a gene or a group of genes, can distinguish between patients who have cancer and those who do not. The AUC values at 1, 2, and 3 years (0.69, 0.66, and 0.67, respectively) indicated the potential of LRrisk to predict overall survival in NSCLC patients within the TCGA datasets (Fig. 4H). Validation of the model employed additional patient cohorts from GSE31210, GSE50081, and GSE3134, which corroborated the findings (Fig. 4I-N).

Assessment of factors influencing NSCLC patient survival in the TCGA dataset revealed significant differences in molecular subtypes, age, T stages, and pathological stages that were observed when stratified by risk scores, with higher scores correlating with more advanced malignancy (Fig. S2A–D). Pie charts illustrating the results of the chi-square test for clinicopathological factors and LRrisk subgroups within each LRrisk score category were generated (Fig. S2E). Univariate Cox regression showed high-risk scores were associated with poor prognosis (Fig. S2F), and multivariate analysis confirmed LRrisk, along with age and stage factors, as independent prognostic indicators (Fig. S2G). A prognostic nomogram integrating LRrisk and clinical stage accurately predicted patient survival (Fig. S2H), validated by precise calibration curves (Fig. S2I) and Decision Curve Analysis (Fig. S2J), affirming the independent prognostic value of LRrisk. Compared with other clinicopathological features, the LRrisk exhibited a more powerful capacity for survival prediction (Fig. S2K).

### Correlation between LRrisk and immune cell infiltration or immunotherapy response in NSCLC

**Fig. 5:**
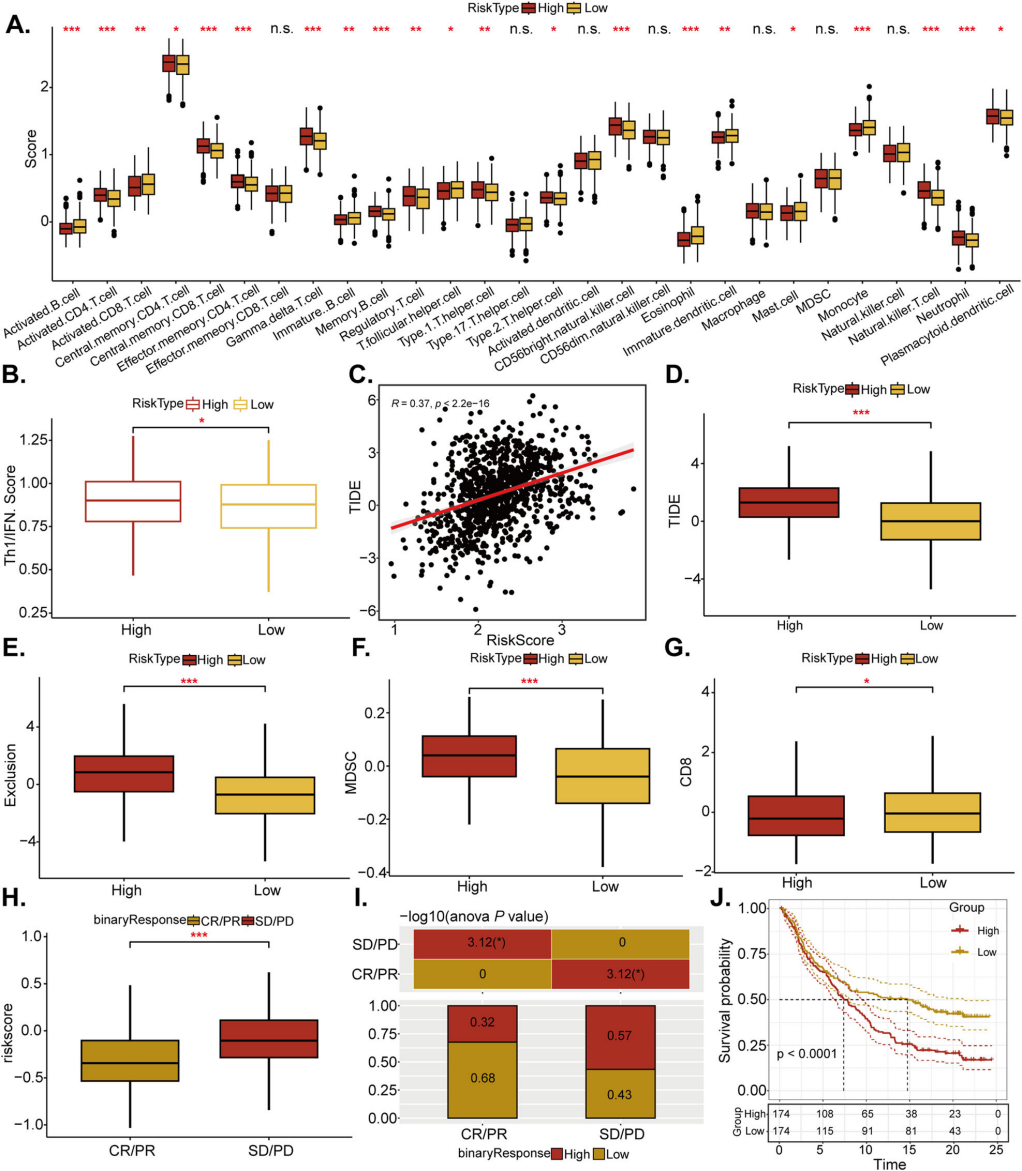
Correlation between LRrisk and immune cell infiltration or immunotherapy response in NSCLC. Analysis of the immune cell scores between the high-risk and low-risk groups using the CIBERSORT algorithm (**A**). The Th1/IFN Score, indicative of a Th1/IFN-gamma response, was compared between the groups (**B**). A correlation analysis was conducted to examine the relationship between the TIDE score and the risk-score (**C**). Further TIDE analysis within the TCGA-NSCLC cohort revealed differences in TIDE scores (**D**), measures of immune exclusion (**E**), the presence of MDSC (**F**), and CD8 + T cell expression (**G**) between the two groups. The LRrisk score’s impact on patient response to immunotherapy, categorized by CR/PR and SD/PD, was analyzed in the IMvigor210 cohort (**H**). The proportion of SD/PD outcomes post-immunotherapy was compared to patients with high versus low-risk scores (**I**). KM analysis was employed to delineate the prognostic significance of the LRrisk score in IMvigor210 cohort (**J**). *P < 0.05; **P < 0.01; ***P < 0.001; “n.s.” stands for *P* > 0.05.

The distribution of 28 immune cells within the TCGA-NSCLC cohort was analyzed, along with the differences between risk score groups. Significant variations in immune cell infiltration levels were observed among patients (Fig. 5A), with these levels displaying notable differences between distinct risk-score groups. Specifically, CD4^+^ T cells were found to infiltrate significantly, exhibiting enhanced infiltration observed in the high-risk score group when compared to the low-risk score group. Additionally, the high-risk group showed a significantly higher Th1/IFN-γ score compared to the low-risk group (Fig. 5B). A positive linear correlation was also identified between the risk and TIDE scores (Fig. 5C), indicating a direct association between high-risk group ratings and increased TIDE score, exclusion score, and MDSC expression (Fig. 5D–F). Conversely, the low-risk group demonstrated a correlation with a rise in CD8 expression (Fig. 5G). In the absence of specific NSCLC patient data for immune checkpoint inhibitor treatment, we analyzed datasets from patients with urothelial cancer who received anti-PD-1 therapy. The analysis demonstrated that a greater percentage of patients with a high-risk score experienced either stable disease or disease progression following immunotherapy in contrast to the low-risk score group (64% versus 28%) (Fig. 5H and I). Kaplan–Meier survival analysis further indicated that patients with a high-risk score were more likely to have poorer survival outcomes (Fig. 5J). These findings imply that the risk score model might serve as a valuable indicator for predicting the efficacy of immunotherapy.

### Expression of 12 different epithelial ligand-receptor interaction-related genes in NSCLC cells and normal lung epithelial cells

**Fig. 6:**
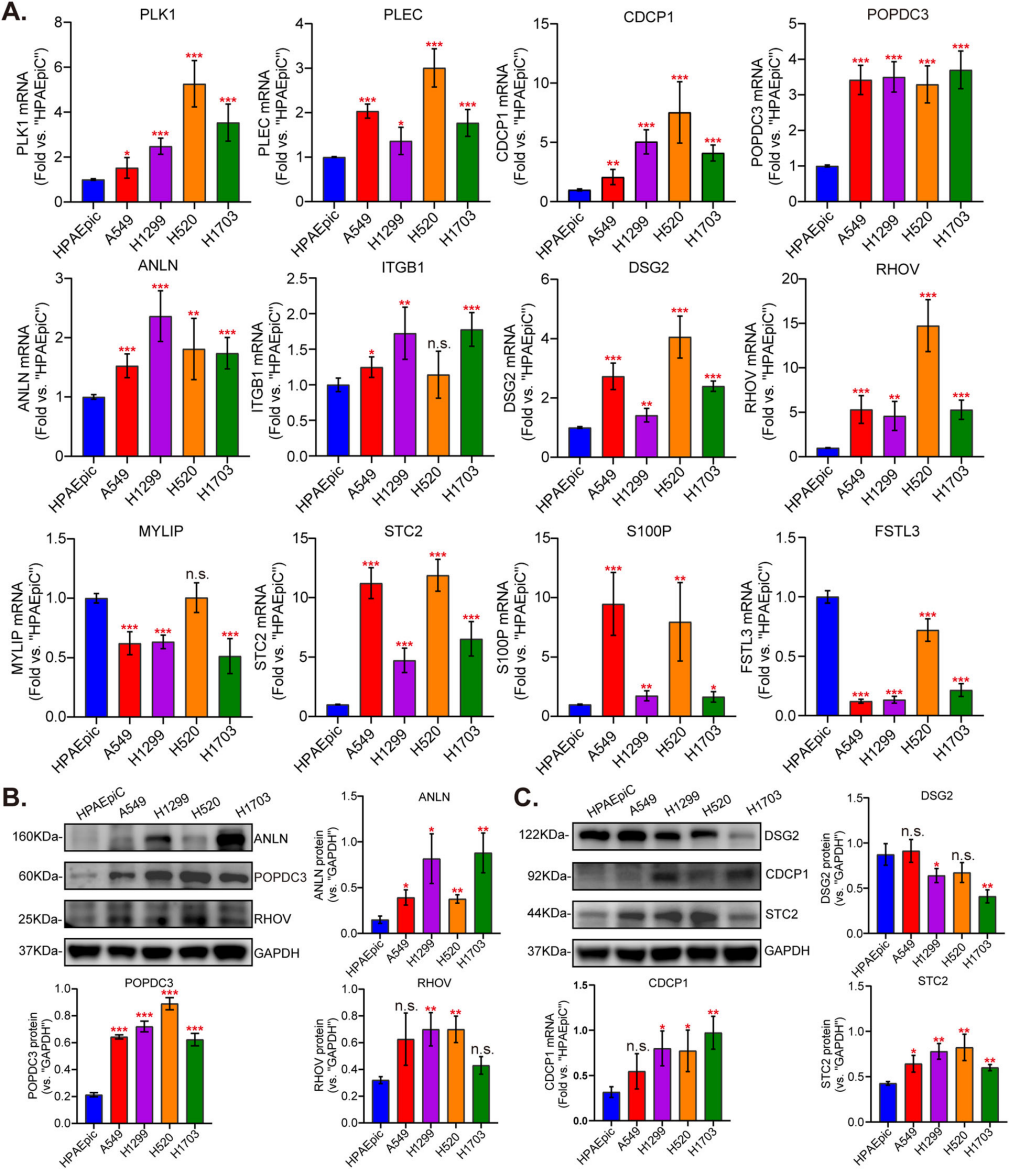
Expression of 12 different epithelial ligand-receptor interaction-related genes in NSCLC cells and normal lung epithelial cells. qRT-PCR was utilized to measure mRNA expression in a human lung epithelial cell line alongside four established NSCLC cells (A549, H1299, H520, and H1703) (**A**). The levels of listed proteins were examined (**B**, **C**). *P < 0.05; **P < 0.01; ***P < 0.001; “n.s.” stands for *P* > 0.05.

The model demonstrated a pronounced correlation with NSCLC, suggesting that the independent prognostic genes could fundamentally affect the biological functions of NSCLC cells. Consequently, we hypothesized that the overexpressed genes identified in our model would be of considerable interest for further investigation. The validation of mRNA expression was conducted by performing qRT-PCR on human lung epithelial cells (HPAEpic) as well as four distinct NSCLC cell lines, including A549, H1299, H520, and H1703 (Fig. 6A). To assess the protein expression of POPDC3, CDCP1, DSG2, and RHOV, we utilized Western blotting assays. The results confirmed an upregulation of these proteins in the established NSCLC cells (Fig. 6B, C). Among all these tested genes, POPDC3 (popeye domain-containing protein 3) emerged as a significant molecule with markedly high expression in all the tested immortalized NSCLC cells (Fig. 6B, C).

### Expression and prognostic relevance of POPDC3 in NSCLC

**Fig. 7:**
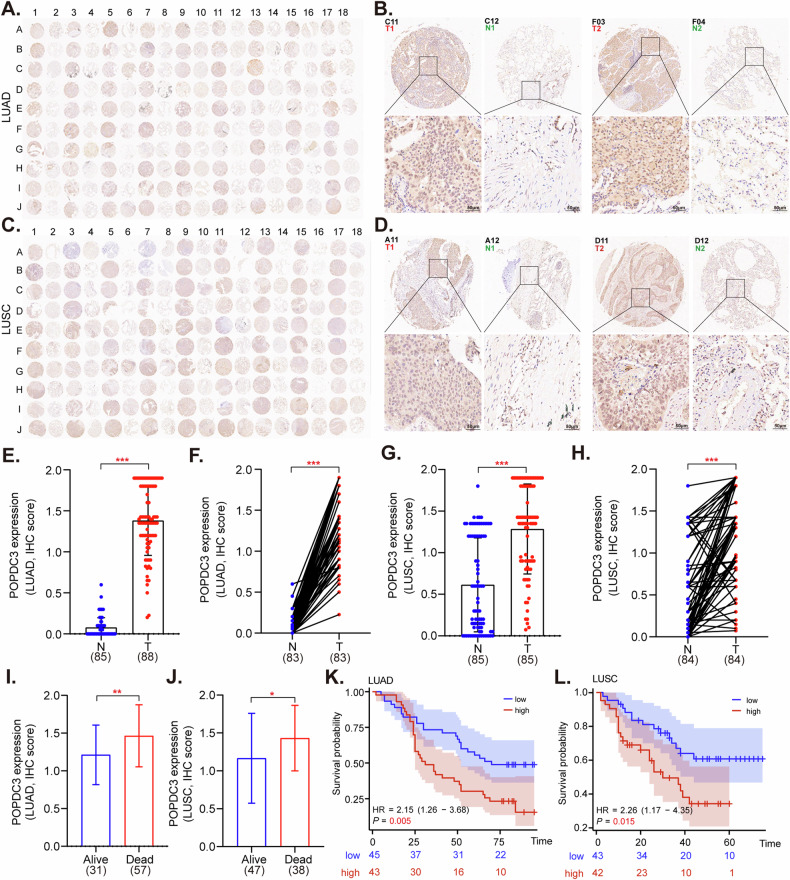
Expression and prognostic relevance of POPDC3 in NSCLC. POPDC3 immunohistochemistry (IHC) microarray of NSCLC tissues and their adjacent normal tissues were shown (**A**, **C**) and the representative IHC images of each microarray were presented (**B**, **D**). The expression of POPDC3 was quantified and compared in LUAD and LUSC tissues versus adjacent normal tissues, analyzed both as unpaired and paired samples (**E**–**H**). The association between POPDC3 intensity scores and OS events was investigated for both LUAD (**I**) and LUSC (**J**) cases. KM survival analyses based on POPDC3 expression in LUAD patients (**K**) and LUSC patients (**L**) were shown. *P < 0.05; **P < 0.01; ***P < 0.001; “n.s.” stands for *P* > 0.05. Scale bar = 100 μm.

POPDC3 is a Popeye domain-containing family protein and is primarily involved in signaling pathways critical for cell communication [37, 38]. This protein family is mainly expressed in cardiac and skeletal muscle tissues, but its expression and potential functions in human cancer are largely unknown [37, 38]. IHC analysis of POPDC3 was conducted on two NSCLC tissue microarrays (TMAs), comprising 90 samples of lung adenocarcinoma (LUAD) (Fig. 7A, B) and 90 samples of lung squamous cell carcinoma (LUSC) (Fig. 7C, D), along with corresponding adjacent non-cancerous tissue samples. The analysis revealed a notable overexpression of POPDC3 in NSCLC tissues compared to adjacent non-cancerous tissues (*P* < 0.05, Fig. 7E–H). Additionally, elevated expression of POPDC3 was significantly associated with adverse clinical outcomes (*P* < 0.05, Fig. 7I, J) and poorer survival of NSCLC patients (*P* < 0.05, Fig. 7K, L).

Integration of the LUAD and LUSC groups into a consolidated NSCLC cohort reaffirmed the enhanced expression of POPDC3 in cancerous tissues (*P* < 0.05, Fig. S3A, B). Intriguingly, while no significant associations were found between POPDC3 expression and patient gender (Fig. S3C), age (Fig. S3D), T stage (Fig. S3E**)**, and clinical stage (Fig. S3F), where a strong correlation was observed with the occurrence of overall survival events (Fig. S3G), N stage (Fig. S3H), and pathological grade (Fig. S3I). Kaplan–Meier plots further verified that higher POPDC3 expression correlates with reduced overall survival (*P* < 0.001, Fig. S3J). Subgroup analyses via Kaplan–Meier curves indicated that patients in the high-risk group bounded by median POPDC3 expression, exhibited significantly lower overall survival rates across various clinical strata, including T1 or T3&T4, N0 or N1&2&3, stage III–IV, and G1-2 classifications (Fig. S3K–N). These findings suggest that POPDC3 holds considerable promise as a prognostic indicator in NSCLC.

### POPDC3 silencing inhibits NSCLC cell proliferation, motility, invasion, and epithelial-mesenchymal transition (EMT)

**Fig. 8:**
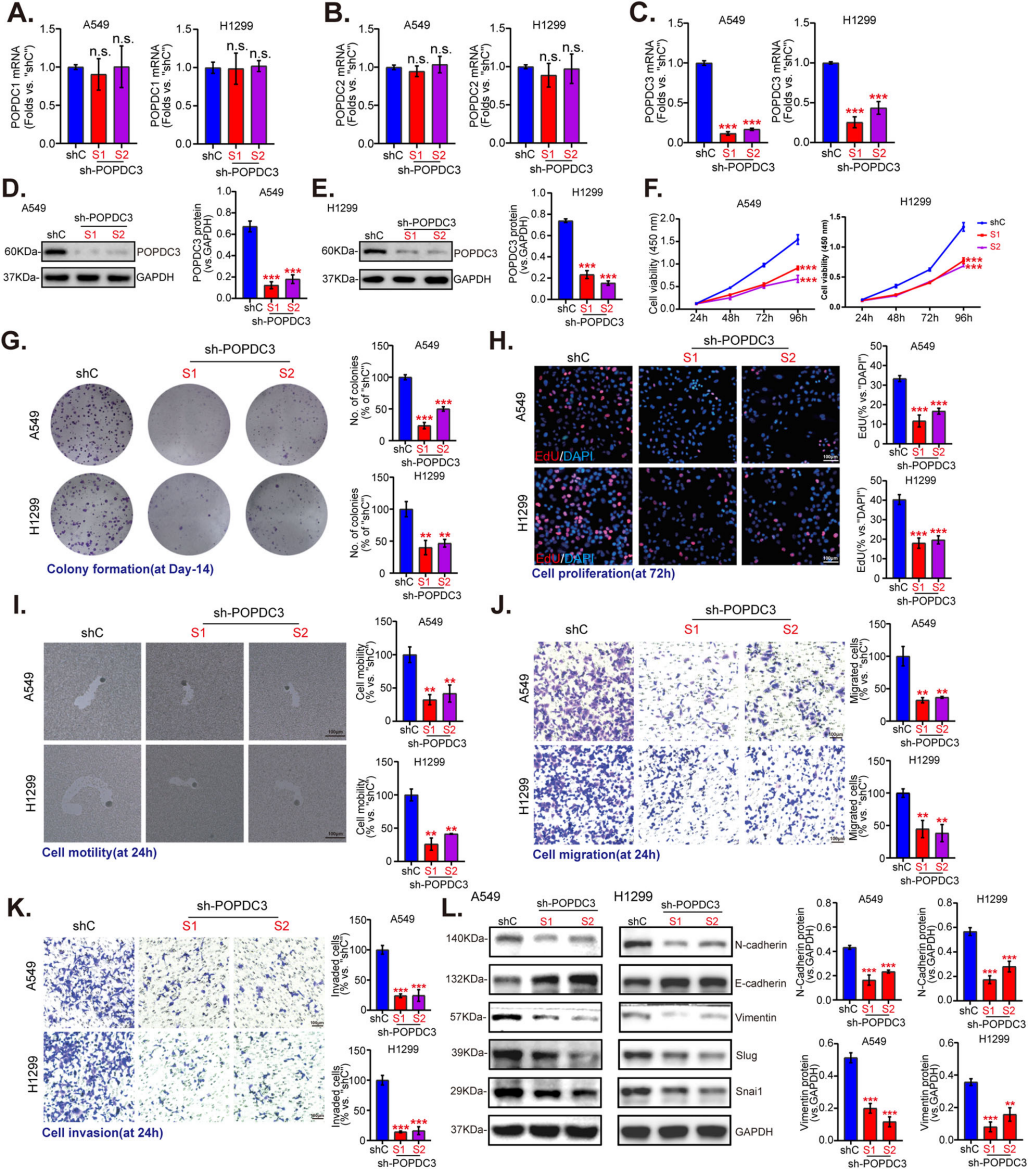
POPDC3 silencing inhibits NSCLC cell proliferation, motility, invasion, and epithelial-mesenchymal transition (EMT). Established NSCLC cell lines (A549, H1299) were genetically modified using shRNA sequences targeting POPDC3 (“sh-POPDC3 -S1/S2”) or a non-targeting scramble control shRNA (“shc”). Cells were then cultivated and screened for changes in the expression of listed genes and proteins (**A**–**E**); The impact of POPDC3 suppression on cell behaviors was assessed over indicated cultivation durations, examining cell viability (**F**), colony-forming capabilities (**G**), EdU incorporation (**H**), and cell motility (**I**). The cell migration (**J**) and invasion (**K**) were evaluated with established assays. The expression of listed proteins was analyzed using Western blotting (**L**). Error bars stand for mean ± standard deviation (SD, *n* = 3). Statistical significance is denoted as *P < 0.05; **P < 0.01; ***P < 0.001 versus “shc” cells, while “n.s.” stands for *P* > 0.05. Scale bar = 100 μm.

Lentivirus expressing POPDC3-targeting shRNA (“POPDC3-sh-S1/S2”, targeting non-overlapping sequences) was transduced into established NSCLC A549, H1299 cell lines and primary human NSCLC cells (“priNSCLC-1”), the mRNA expression levels of control genes, POPDC1 and POPDC2, remained unchanged upon POPDC3 silencing (Figs. 8A, B and S4A, B). There is however a marked decrease in *POPDC3* mRNA and protein levels, as verified by qRT-PCR (Fig. 8C, Fig. S4C) and Western blotting assay (Fig. 8D, E, Fig. S4D). CCK-8 assay results demonstrated a significant decrease in NSCLC cell viability upon POPDC3 knockdown (Figs. 8F and S4E). Colony formation assays revealed a substantial reduction in the numbers of A549, H1299, and priNSCLC-1 cell colonies following POPDC3 silencing (Figs. 8G and S4F). Moreover, POPDC3 shRNA hindered NSCLC cell proliferation, as evidenced by a significant reduction in EdU-positive nuclei ratio (Figs. 8H and S4G). Phagokinetic track motility assay findings supported that the silencing of POPDC3 impeded the motility of NSCLC cells (Fig. 8I). shRNA-induced silencing of POPDC3 also resulted in inhibition of cell migration (Figs. 8J and S4H) and invasion (Figs. 8K and S4I). Moreover, POPDC3 shRNA led to the upregulation of the epithelial marker E-cadherin and decreased levels of mesenchymal markers, including N-cadherin, and vimentin, as well as the epithelial-mesenchymal transition (EMT) transcription factors, Slug and Snai1 (Figs. 8L and S4J), indicating its possible role in EMT of NSCLC. Therefore, POPDC3 shRNA inhibited cell proliferation, motility, invasion, and EMT in NSCLC cells.

### POPDC3 overexpression exerts pro-cancerous activity in NSCLC cells

**Fig. 9:**
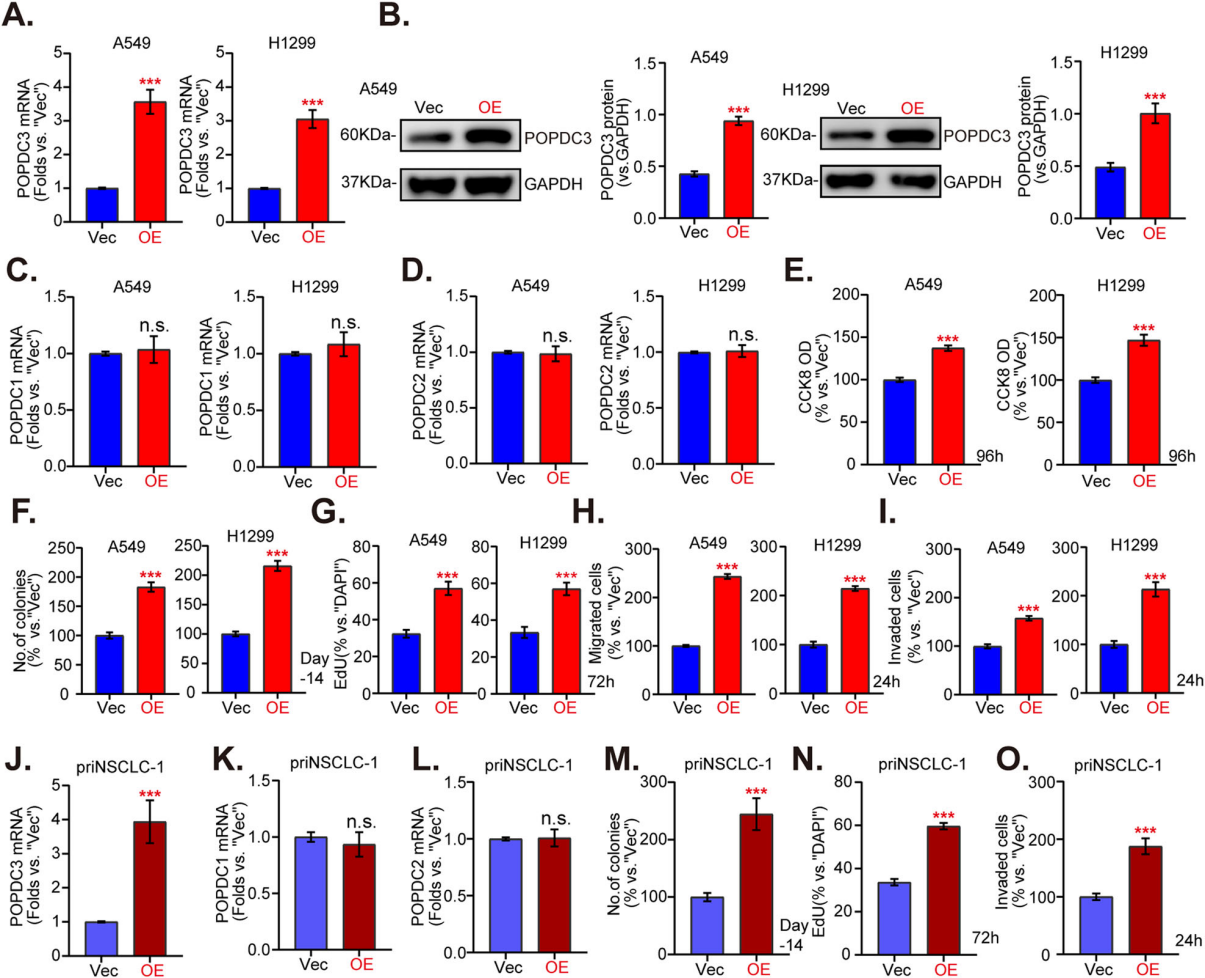
POPDC3 overexpression exerts pro-cancerous activity in NSCLC cells. Established NSCLC cell lines (A549, H1299) (**A**–**I**) along with primary human NSCLC cells (priNSCLC-1) (**J**–**O**) were genetically modified to overexpress POPDC3 via a lentiviral vector (“POPDC3-OE”) compared to a control vector (“Vec”), employing puromycin selection. The expression of listed genes and proteins was shown (**A**–**D**, **J**–**L**). Subsequently, after a designated incubation period, the cells underwent various assays to assess cell viability (**E**), cell colony formation (**F**, **M**), cell proliferation indicated by EdU incorporation (**G**, **N**), as well as migration (**H**) and invasion (**I**, **O**). Error bars stand for mean ± standard deviation (SD, *n* = 3). Statistical significance is denoted as *P < 0.05; **P < 0.01; ***P < 0.001 versus “Vec” cells, while “n.s.” stands for *P* > 0.05. Scale bar = 100 μm.

We investigated the potential pro-cancerous activity of POPDC3 through ectopic overexpression in A549 and H1299 cells. NSCLC cells were infected with a lentiviral PODC3-expressing construct, and following selection using puromycin-containing medium, POPDC3-overexpressed NSCLC cells, “POPDC3-OE”, were formed. These POPDC3-OE cells exhibited a significant increase in POPDC3 expression at both the mRNA (Fig. 9A) and protein (Fig. 9B) levels, without affecting the expression levels of POPDC1 and POPDC2 (Fig. 9C, D). Ectopic overexpression of POPDC3 further promoted cell viability, as indicated by the CCK-8 assay optical density (Fig. 9E), cell colony formation (Fig. 9F), and cell proliferation (evidenced by elevated EdU incorporation rates, Fig. 9G) of NSCLC cells. Additionally, POPDC3-OE NSCLC cells demonstrated accelerated in vitro migration (Fig. 9H) and invasion (Fig. 9I). In the primary NSCLC cells, priNSCLC-1, stable transfection of the OE-POPDC3 construct led to robust *POPDC3* mRNA and protein upregulation (Fig. 9J), where POPDC1 and POPDC2 mRNA levels were unchanged (Fig. 9K, L). In these primary cancer cells, ectopic overexpression of POPDC3 augmented cell colony formation (Fig. 9M), cell proliferation (increased EdU-positive nuclei ratio, Fig. 9N), migration, and invasion (“Matrigel Transwell assays”, results quantified in Fig. 9O). These findings suggest that POPDC3 overexpression exerted pro-cancerous activity in NSCLC cells in vitro.

### POPDC3 shRNA suppresses H1299 xenograft growth in vivo

**Fig. 10:**
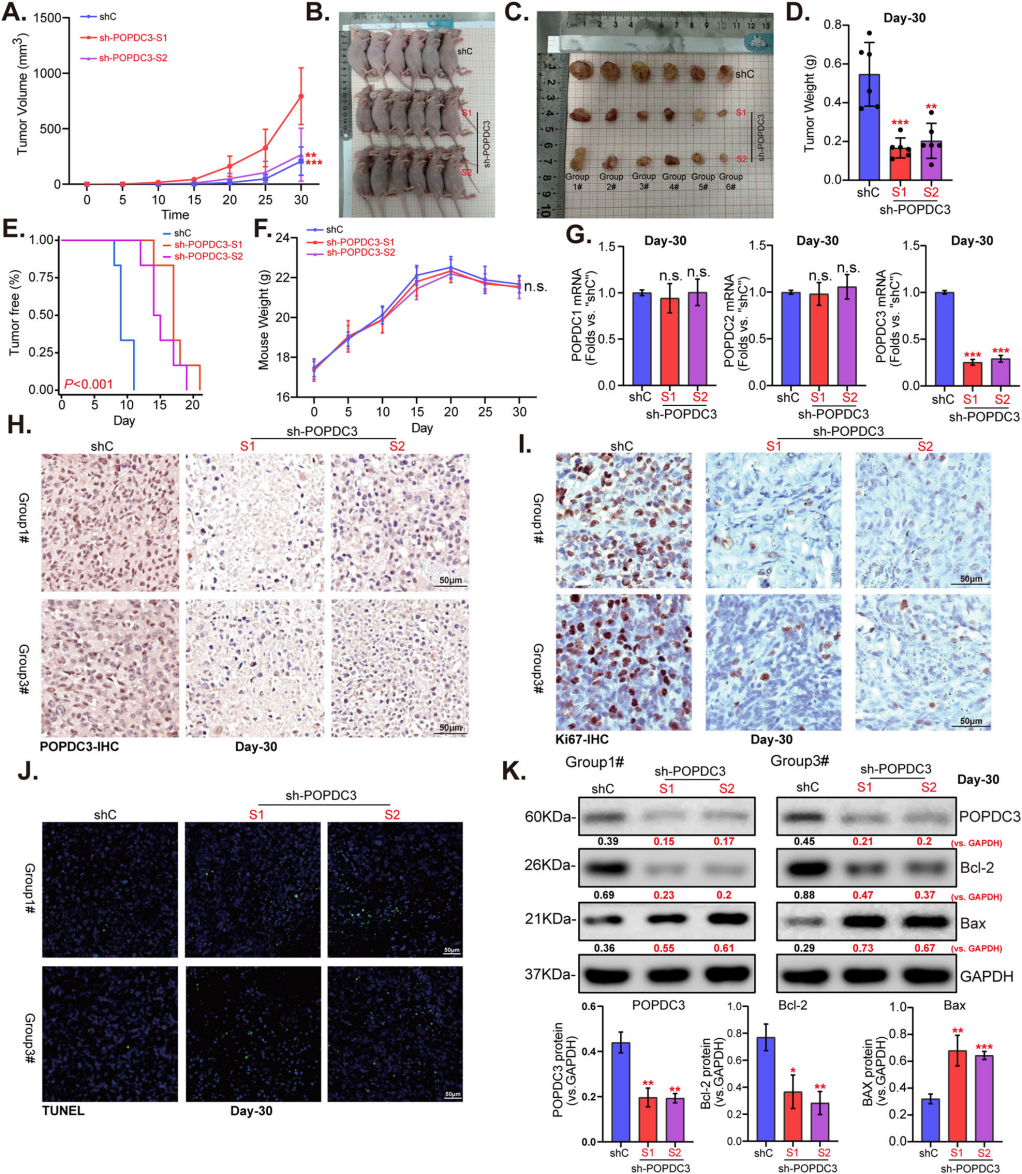
POPDC3 shRNA suppresses H1299 xenograft growth in vivo. Female BALB/c nude mice were implanted with H1299 cells expressing POPDC3-targeting shRNA (“POPDC3-sh-S1”, “POPDC3-sh-S2”) or a non-targeting scramble control shRNA (“shC”) to establish the experimental model. Tumor growth was monitored by measuring tumor volumes (**A**) and recording mouse body weights (**F**) at 5-day intervals. After 30 days, tumors were surgically excised (**B**, **C**), and their weights were measured (**D**). A tumor-free survival curve was also displayed (**E**). Within the harvested tumor tissues, the levels of *listed genes* (**G**) were evaluated, with results quantified. Representative IHC images of POPDC3 (**H**) and Ki67 (**I**) in tumor tissues were shown. Additionally, representative images of TUNEL staining on tumor tissues were presented (**J**). The expression of apoptosis-related proteins was analyzed using Western blotting (**K**). Statistical significance is denoted as *P < 0.05; **P < 0.01; ***P < 0.001 versus “shc” cells, while “n.s.” stands for *P* > 0.05. Scale bar = 50 μm.

To estimate the influence of POPDC3 on NSCLC cell growth in vivo. H1299 cells bearing “sh-POPDC3” or “shC” were subcutaneously (s.c.) injected into the right flank of nude mice. Each group consisted of six mice, with each mouse receiving an inoculation of three million cells. Tumor growth was monitored by measuring the tumor volumes every 5 days from the initial day of injection (Day 0) until Day 30 (Fig. 10A). The growth curves revealed a significant reduction in the volume of sh-POPDC3-expressing H1299 xenografts compared to shC control group (Fig. 10B). At Day 30, all mice were euthanized, tumors were harvested, and their weights were measured. The sh-POPDC3 H1299 xenografts were considerably smaller (Fig. 10C) and lighter (Fig. 10D) compared to the shC control group. The NSCLC xenograft model also exhibited pronounced delays in tumor development upon POPDC3 silencing (Fig. 10E), with no significant differences observed in the body weights of the mice (Fig. 10F).

Analysis of fresh tumor tissue lysates revealed substantial reductions in *POPDC3* mRNA and protein levels in sh-POPDC3 xenografts (Fig. 10G, K), while the mRNA levels of POPDC1 and POPDC2 remained unchanged (Fig. 10G). Consistent with in vitro observations, IHC staining of the sh-POPDC3 xenograft sections confirmed dramatic POPDC3 silencing (Fig. 10H). Decreased Ki-67 staining in the sh-POPDC3 H1299 xenograft sections further indicated reduced cell proliferation in vivo (Fig. 10I). Enhanced apoptosis was indicated by an increase in the ratio of TUNEL-positive nuclei (Fig. 10J) and changes in the expression of apoptosis-related proteins, with an upregulation of Bax and a downregulation of BCL-2 (Fig. 10K). These findings support that POPDC3 silencing inhibited the growth of H1299 xenografts in nude mice.

### PODPC3 overexpression promotes primary NSCLC cell growth in vivo

**Fig. 11:**
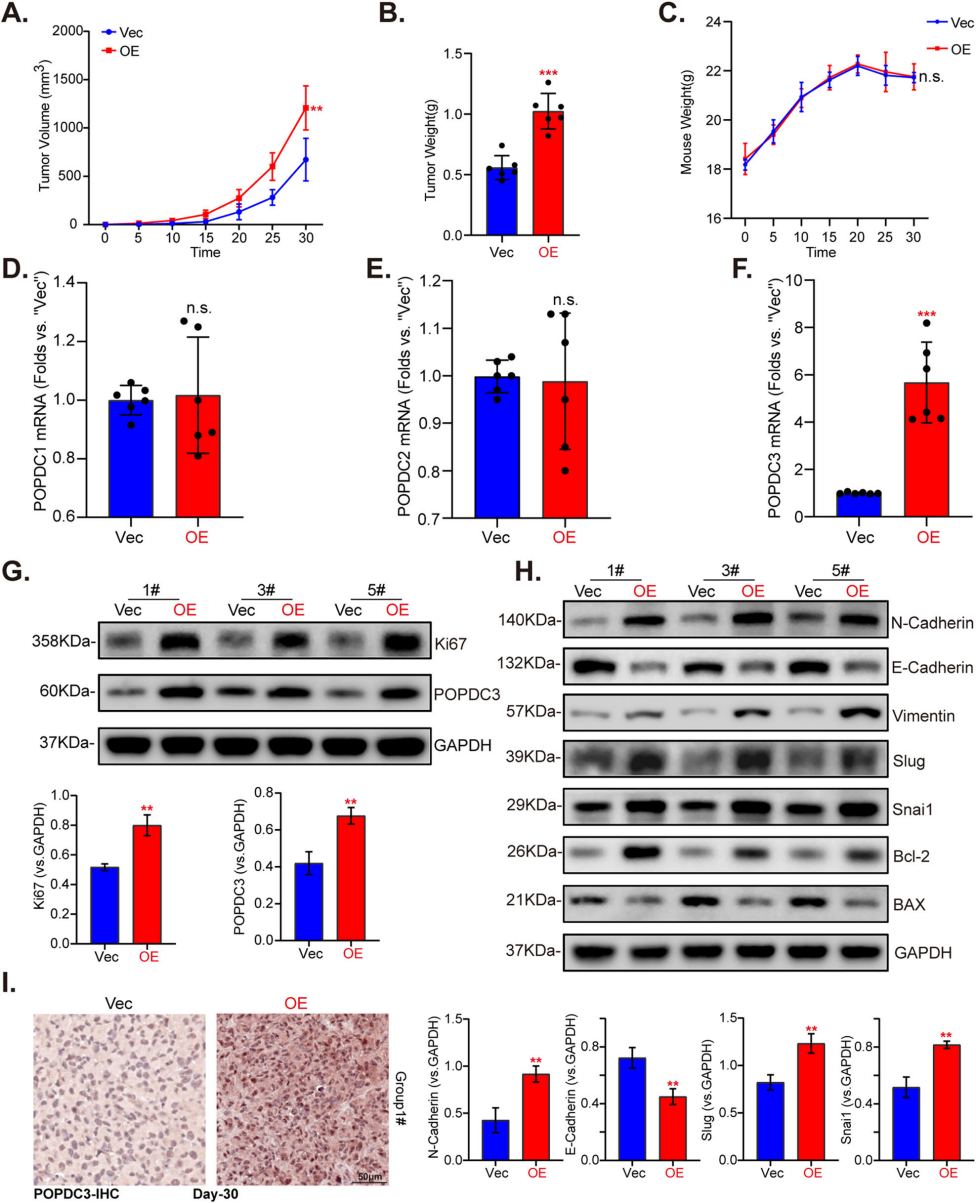
PODPC3 overexpression promotes primary NSCLC cell growth in vivo. At 3 × 10^6^ cells per mouse, priNSCLC-1 primary NSCLC cells with the lentiviral POPDC3 overexpression construct (“POPDC3-OE”) or the corresponding vector (“Vec”) were s.c. injected to the nude mice to establish the experimental model. Tumor growth was monitored by measuring tumor volumes (**A**) and recording mouse body weights (**C**) at 5-day intervals. After 30 days, tumors were surgically excised, and their weights were measured (**B**). Within the harvested tumor tissues, the levels of listed genes and proteins (**D–H**) were evaluated, with results quantified. Representative IHC images of POPDC3 in tumor tissues were shown (**I**). Data were presented as mean ± standard deviation (SD). Statistical significance is denoted as *P < 0.05; **P < 0.01; ***P < 0.001 versus “Vec” cells, while “n.s.” stands for *P* > 0.05. Scale bar = 50 μm.

Next, we assessed the effect of POPDC3 overexpression on NSCLC cell growth in vivo. The priNSCLC-1 cells with the lentiviral POPDC3 overexpression construct (“POPDC3-OE”) or the corresponding vector (“Vec”) were *s.c*. injected into the flanks of the nude mice. Tumor growth was tracked by measuring tumor volumes every 5 days from the day of injection (Day 0) until Day 30 (Fig. 11A). The results showed that POPDC3-OE priNSCLC-1 xenografts were heavier in weight (Fig. 11B) compared to the Vec group. There were no significant differences in the body weights of the mice between the two groups (Fig. 11C). Analysis of fresh tumor tissue lysates revealed the mRNA levels of POPDC1 and POPDC2 remained unchanged (Fig. 11D, E), but there was a substantial upregulation in *POPDC3* mRNA and protein levels in POPDC3-OE priNSCLC-1 xenografts (Fig. 11F, G). Ki-67 protein expression increased, indicating enhanced proliferation (Fig. 11G). Moreover, changes of EMT-related proteins, including upregulation of N-Cadherin, Vimentin, Slug, but downregulation of N-Cadherin were detected in POPDC3-OE priNSCLC-1 xenograft tissues (Fig. 11H), where Bcl-2 levels were increased, and BAX levels were decreased (Fig. 11H). IHC staining further confirmed POPDC3 overexpression in POPDC3-OE priNSCLC-1 xenograft sections (Fig. 11I). These results further support that POPDC3 overexpression promoted NSCLC cell growth in vivo.

### POPDC3 overexpression is associated with immune cell infiltration in NSCLC

**Fig. 12:**
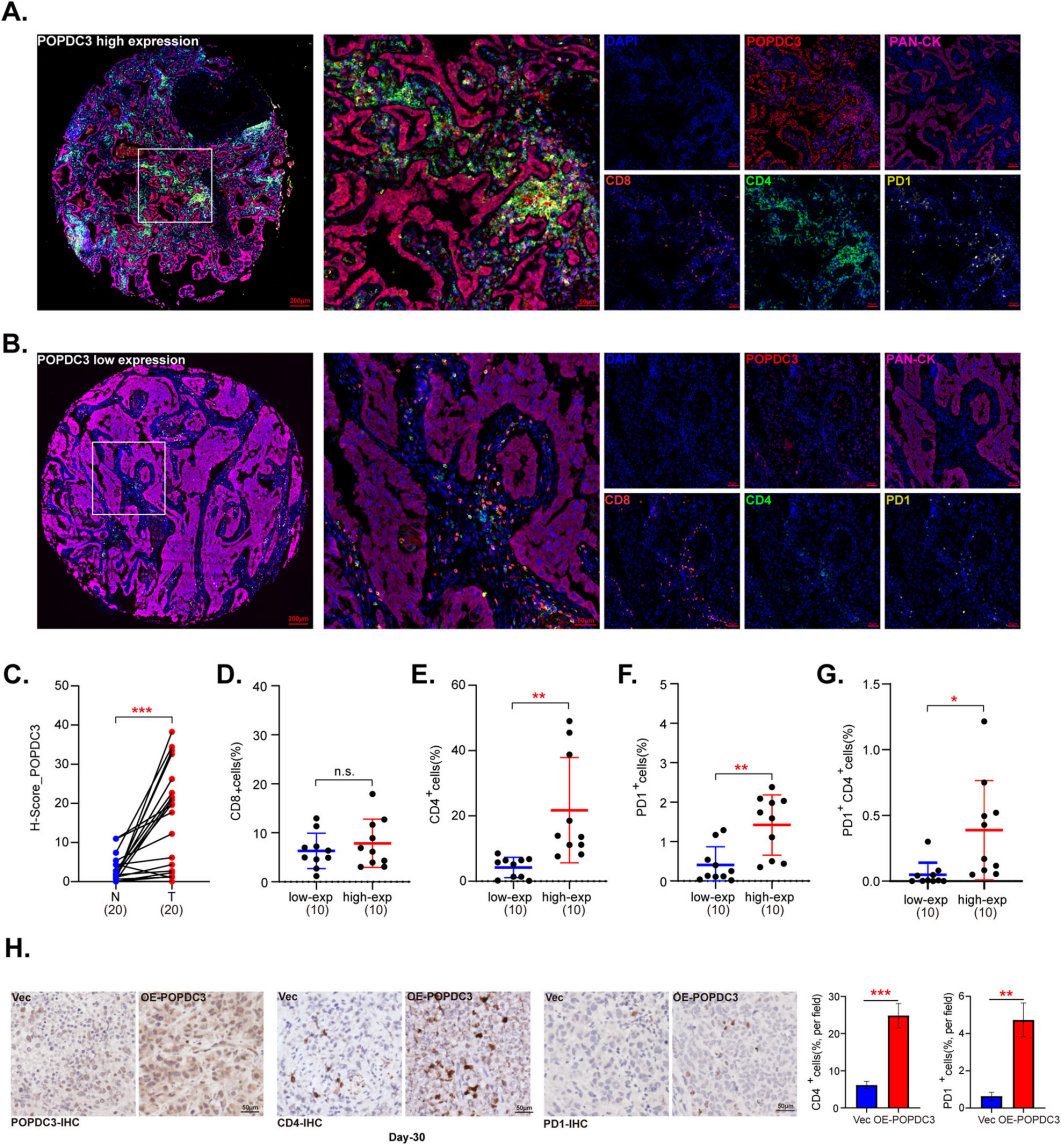
POPDC3 overexpression is associated with immune cell infiltration in NSCLC. Representative mIHC images of patient samples with high-POPDC3 expression (**A**) and low-POPDC3 expression (**B**) were presented, with each color panel shown alongside. Various markers were visualized by distinct colors, including POPDC3 in red, CD8 in turquoise, CD4 in green, Pan-CK in pink and PD1 in gold, with DAPI used for counterstaining. Quantitative comparison of POPDC3 distribution within NSCLC tissue relative to normal tissue was conducted using an H-score (**C**). The association between POPDC3 expression levels and the presence of CD8^+^ (**D**) and CD4^+^ T cells (**E**) was investigated. The proportion of PD1^+^ cells (**F**) and CD4^+^PD1^+^ cells (**G**) was explored by comparing the high versus low-POPDC3-expressing groups within the tumor tissues (**F**). At 3 × 10^6^ cells per mouse, Lewis lung carcinoma (LLC) cells with the lentiviral POPDC3 overexpression construct (“POPDC3-OE”) or the corresponding vector (“Vec”) were *s.c*. injected to the C57BL/6 J mice to establish the experimental model. After 30 days, the mice were sacrificed, and the NSCLC tumor tissues were fixed with 10% formaldehyde, sectioned and analyzed by immunohistochemical staining (**G**). Representative IHC staining of POPDC3, CD4 and PD1, and their quantitative analyses in LLC xenograft tissues in C57BL/6J mice (**H**). *P < 0.05; **P < 0.01; ***P < 0.001; “n.s.” stands for *P* > 0.05. Scale bar = 50 μm.

Our abovementioned results indicate that the LRrisk scoring model may serve as a predictive biomarker for immunotherapy of NSCLC, within which POPDC3 is significantly overexpressed in NSCLC and correlates with poor prognosis of NSCLC patients. To further investigate the role of POPDC3, identified as a key LRG, in modulating immune responses within the tumor microenvironment, we employed multiplex immunohistochemistry (mIHC). This approach enabled the simultaneous measurement of five key biomarkers: PAN-CK, POPDC3, CD4, CD8, and PD1 in NSCLC samples (Fig. 12A, B). Utilizing the H-score methodology, we confirmed a higher expression of POPDC3 in NSCLC tissues compared to adjacent normal tissues (Fig. 12C). Subsequently, NSCLC tissues were classified into two groups based on POPDC3 expression levels: “POPDC3-low expression” and “POPDC3-high expression”. Our analysis demonstrated a significant positive correlation between elevated POPDC3 expression and enhanced infiltration of CD4^+^ T cells within the NSCLC tissues (Fig. 12D). However, no similar correlation was observed with CD8^+^ T cell densities (Fig. 12E). Additionally, an elevated proportion of PD-1 positive cells in NSCLC tissues exhibiting high-POPDC3 expression was detected (Fig. 12F, G). Given the established role of PD-1 as a critical immune checkpoint molecule capable of suppressing immune responses [39], its upregulation concurrent with increased POPDC3 expression may suggest a mechanism by which POPDC3-high expression NSLCLC cells evade immune surveillance. To support this notion, ectopic overexpression of POPDC3 in LLC xenograft tissues in C57BL/6 J mice resulted in increased CD4^+^ T cell infiltration, accompanied by heightened PD-1 expression in the tumor microenvironment (Fig. 12H). This highlights the possible dual role of POPDC3 in promoting T cell recruitment while also contributing to subsequent T cell exhaustion.

## Discussion

Advancements in single-cell RNA sequencing (scRNA-seq) and other technologies have enriched our understanding of the tumor microenvironment (TME) by revealing its cellular diversity and the functional states of these cells [40]. It has significantly advanced our comprehension of cellular heterogeneity in the context of cancer [40]. Beyond simply cataloging cellular constituents, it is essential to elucidate the complex intercellular communication networks crucial for cancer initiation and progression [41–43]. In this study, scRNA-seq analysis delineated eight distinct cell populations within the NSCLC TME, highlighting its compositional complexity. Crucially, these populations interact predominantly via ligand-receptor (LR) interactions [44, 45], a mechanism pivotal in the modulation of tumor behavior and a target for therapeutic intervention. It is exemplified by therapies that inhibit the PD-1/PD-L1 axis to enhance cytotoxic T cell activity and suppress tumor growth, resulting in response rates up to 45% in advanced NSCLC patients [46]. Therefore, the development of targeted therapies that disrupt specific LR interactions, combined with precise patient risk stratification, necessitates an in-depth understanding of the TME, with a particular focus on the cell-cell interactions that modulate tumor behaviors.

Building on these insights, our study presents novel findings. First, we propose a molecular subtyping model for NSCLC based on malignant epithelial-related LR interactions, stratifying patients into three prognostically distinct subtypes (C1, C2, and C3 subtypes). Each subtype is characterized by distinct clinical and pathological features, along with specific activated molecular pathways and immunological profiles. Among these, C1 subtype is associated with a favorable prognosis, while C3 subtype is correlated with a poor outcome. Although previous research has explored prognostic markers within NSCLC, the practical application of these markers has often encountered limitations [47, 48]. By focusing on cell-cell communication within the TME, the current study provides a framework for improved diagnostic and therapeutic strategies.

To predict the prognosis of NSCLC patients, we identified 12 ligand-receptor interaction-related genes (LRGs): including PLK1, ITGB1, ANLN, POPDC3, DSG2, FSTL3, PLEC, STC2, CDCP1, RHOV, S100P, and MYLIP, and we utilized LASSO-Cox regression analysis to construct an LRrisk score. This risk score correlates with disease progression, immune cell infiltration, and response to immunotherapy. The prognostic accuracy of this 12-gene signature was validated across three GEO datasets and an independent TCGA dataset, demonstrating its reliability for predicting overall survival in NSCLC patients. As expected, analysis through the TIDE and scrutiny of the IMvigor cohort reliably indicated that the LRrisk model can serve as an early predictor of immunotherapy and drug responsiveness in NSCLC patients. Here, we established a prognostic risk model incorporating epithelial-related LRs that can forecast the clinical course and treatment response in NSCLC, which has the potential to lead to the development of personalized therapeutic protocols that are specifically tailored to distinct biomarkers.

The twelve identified LRGs are implicated as pivotal factors in the occurrence, progression, and metastasis in NSCLC. For example, PLK1 enhances the stability and transcriptional activity of β-catenin through phosphorylation, facilitating extracellular matrix remodeling crucial for metastatic spread in NSCLC [49]. ANLN plays a crucial role in NSCLC development by activating RHOA and triggering the PI3K/AKT pathway [50]. DSG2 overexpression in NSCLC has been observed, with its knockdown impeding NSCLC growth via the modulation of cell cycle regulators such as p27 and CDK2 [51]. Among the Popeye domain-containing proteins [52], POPDC3 is particularly notable for its extensive expression in mammalian tissues and pronounced expression in skeletal muscle [37, 38]. It is involved in various physiological and pathological processes and its potential as a biomarker and therapeutic target in cancer has been recently suggested [53]. Elevated POPDC3 expression in breast cancer is significantly correlated with HER2^+^ status [54]. In head and neck squamous cell carcinoma (HNSCC), lower POPDC3 expression correlates with longer overall survival [55]. Furthermore, decreased POPDC3 expression in radiation-resistant nasopharyngeal carcinoma tissues correlates with lower progression-free survival [56].

In our study, we have offered valuable insights into the potential roles of overexpressed POPDC3, a protein primarily expressed in cardiac and skeletal muscle tissue under physiological conditions, in the progression of NSCLC cells. However, we recognize that its relatively weak expression in certain NSCLC tissues and cells raises important questions about the physiological relevance of our findings. The elevated POPDC3 expression observed in lentivirus-transfected NSCLC cells may not accurately reflect the conditions found in actual tumors, highlighting the need for caution in interpreting our results. To address this limitation, it is essential for future research to focus on the absolute quantification of POPDC3 in a larger cohort of primary NSCLC tumor samples and cells. Such studies could deepen our understanding of its expression patterns in a physiological context and clarify its potential roles in NSCLC biology. Ultimately, this could inform the development of targeted therapeutic strategies that take into account the nuanced functions of POPDC3 in tumor progression and metastasis.

Our research further reveals that upregulation of POPDC3 is associated with increased CD4^+^ T cell infiltration and elevated PD-1 expression in NSCLC tissues, suggesting a possible interplay between tumor malignancy and the immune response. In NSCLC, heightened CD4^+^ T cell infiltration likely represents an innate immune response to the neoplasm [57], whereas an elevated proportion of PD1-positive cells may indicate immunosuppressive mechanisms within the tumor microenvironment [58]. Tumors employ various strategies, such as upregulating PD-L1 and inducing T cell exhaustion, to evade immune detection and suppression [59, 60]. This phenomenon suggests that although the tumor may trigger a robust immune response, it also acquires the capability to evade immune surveillance and dampen the immune response. Therefore, immune checkpoint inhibitors (such as therapies targeting PD1/PD-L1) are significant in treating such tumors as they can alleviate the inhibitory effects on T cells, restoring their ability to target tumor cells. This also elucidates why certain tumors exhibit a favorable response to immune checkpoint inhibitor treatment, especially those with a substantial infiltration of immune cells.

Our current study primarily focuses on elucidating the role of POPDC3 in NSCLC cells and its correlation with immune cell infiltration. Although we have not directly assessed the efficacy of immunotherapy in this context, our findings indicate a relationship between tumor POPDC3 expression, PD-1-positive CD4^+^ T cell infiltration, and potential T cell exhaustion, which could inform future studies on immunotherapy responsiveness. Yet, further studies are needed to comprehensively evaluate its impact on immunotherapy efficacy, for example evaluating the efficacy of PD-1/PD-L1 antibody in the context of POPDC3 overexpression/silencing in NSCLC xenograft model in C57BL/6 mice. By doing so, we aim to provide a more complete understanding of the interplay between POPDC3 and immunotherapy outcomes in NSCLC.

## Conclusion

We have established a prognostic model utilizing information from the NSCLC cohort in the GEO database for predicting intercellular communication. This model exhibits a significant correlation with tumor immunity and possesses the potential to forecast the efficacy of immunotherapy. Specifically, we have identified POPDC3 as a pivotal gene that contributes to NSCLC cell growth. The molecular signatures associated with intercellular communication provide additional insights for predicting disease progression and prognosis. Our research represents a groundbreaking advancement in guiding personalized treatment approaches for NSCLC patients in future research endeavors.

## Supplementary information

Original data

Supplementary materials

## Data Availability

All data are available upon request.
