# Supplementary material for: A novel identified epithelial ligand-receptor-associated gene signature highlights POPDC3 as a potential therapy target for non-small cell lung cancer
Source: Cell Death Dis. 2025 Feb 19;16(1):114. doi: 10.1038/s41419-025-07410-9 (PMC11840029; doi:10.1038/s41419-025-07410-9)
Supplement: Supplementary file 1 — Original data [file 41419_2025_7410_MOESM1_ESM.pdf]

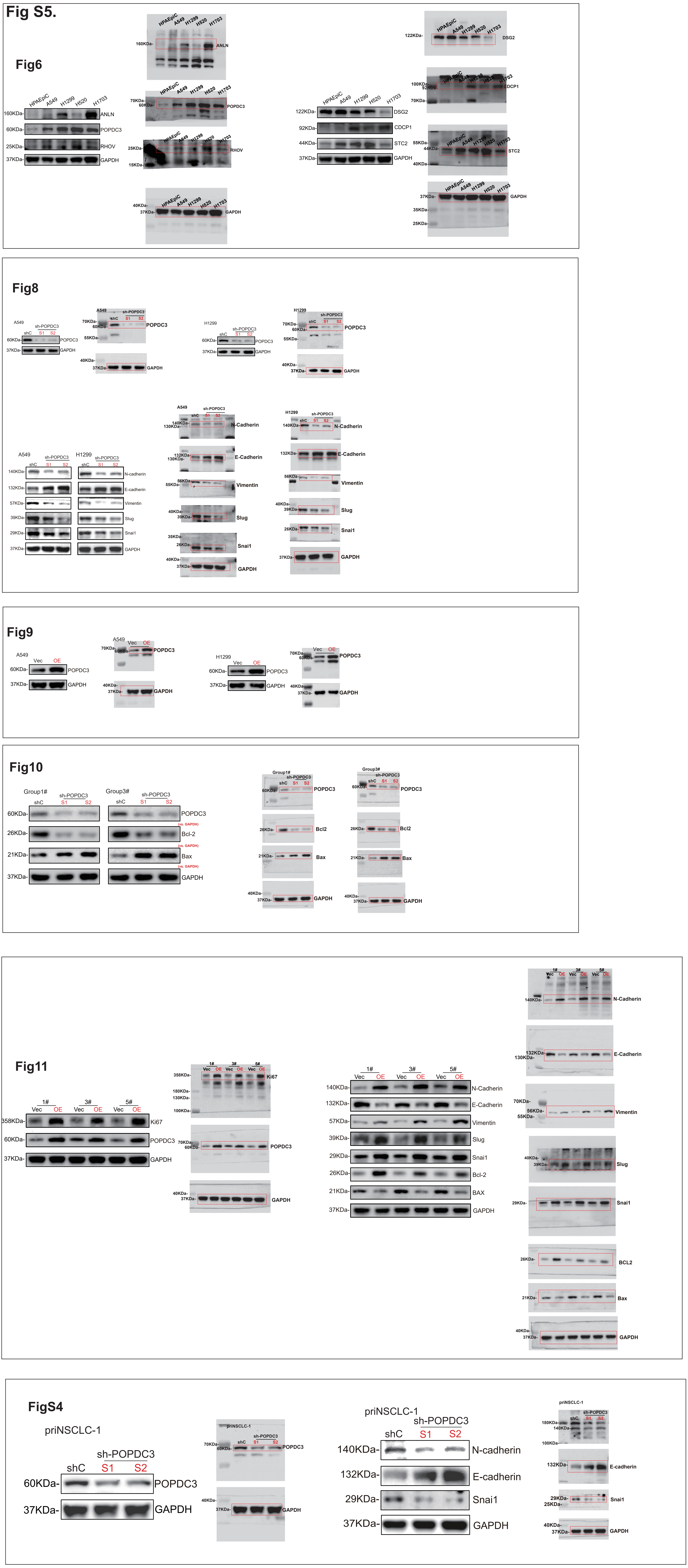

Fig S5: The uncropped blotting images of the study.
The uncropped Western blotting images corresponding to the cropped blots shown in each Figure are presented.
