# Supplementary material for: A novel identified epithelial ligand-receptor-associated gene signature highlights POPDC3 as a potential therapy target for non-small cell lung cancer
Source: Cell Death Dis. 2025 Feb 19;16(1):114. doi: 10.1038/s41419-025-07410-9 (PMC11840029; doi:10.1038/s41419-025-07410-9)
Supplement: Supplementary file 2 — Supplementary materials [file 41419_2025_7410_MOESM2_ESM.pdf]

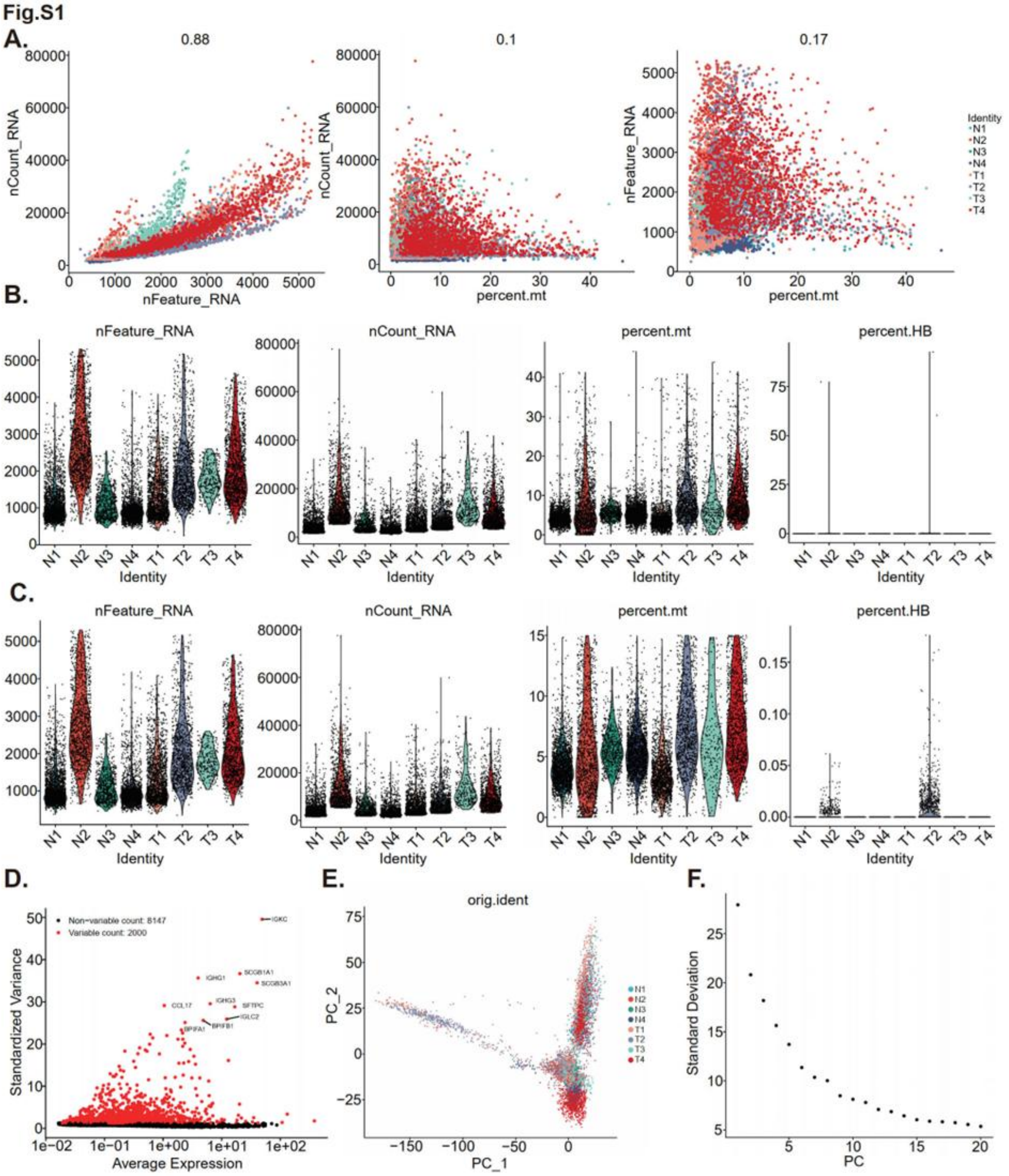

Fig.S1 Single-Cell transcriptome landscape of NSCLC. Dot plot demonstrates the correlation between unique molecular identifier (UMI) counts, mitochondrial genes, and mRNA quantities (**A**). Violin plots demonstrate the sequencing depth, number of detected genes, percentage of mitochondria genome and percentage of erythrocytic genome in each single-cell sample before quality control (**B**) and after quality control (**C**). Dot plot demonstrates the correlation variance of each gene expression across all the single-cell samples. A total of 2,000 genes found to be highly variable are denoted as red dots (**D**). Principal component analysis (PCA) was used to separate cells (**E**). PCA identified 10 principal components (PCs) (**F**).

Fig.S2

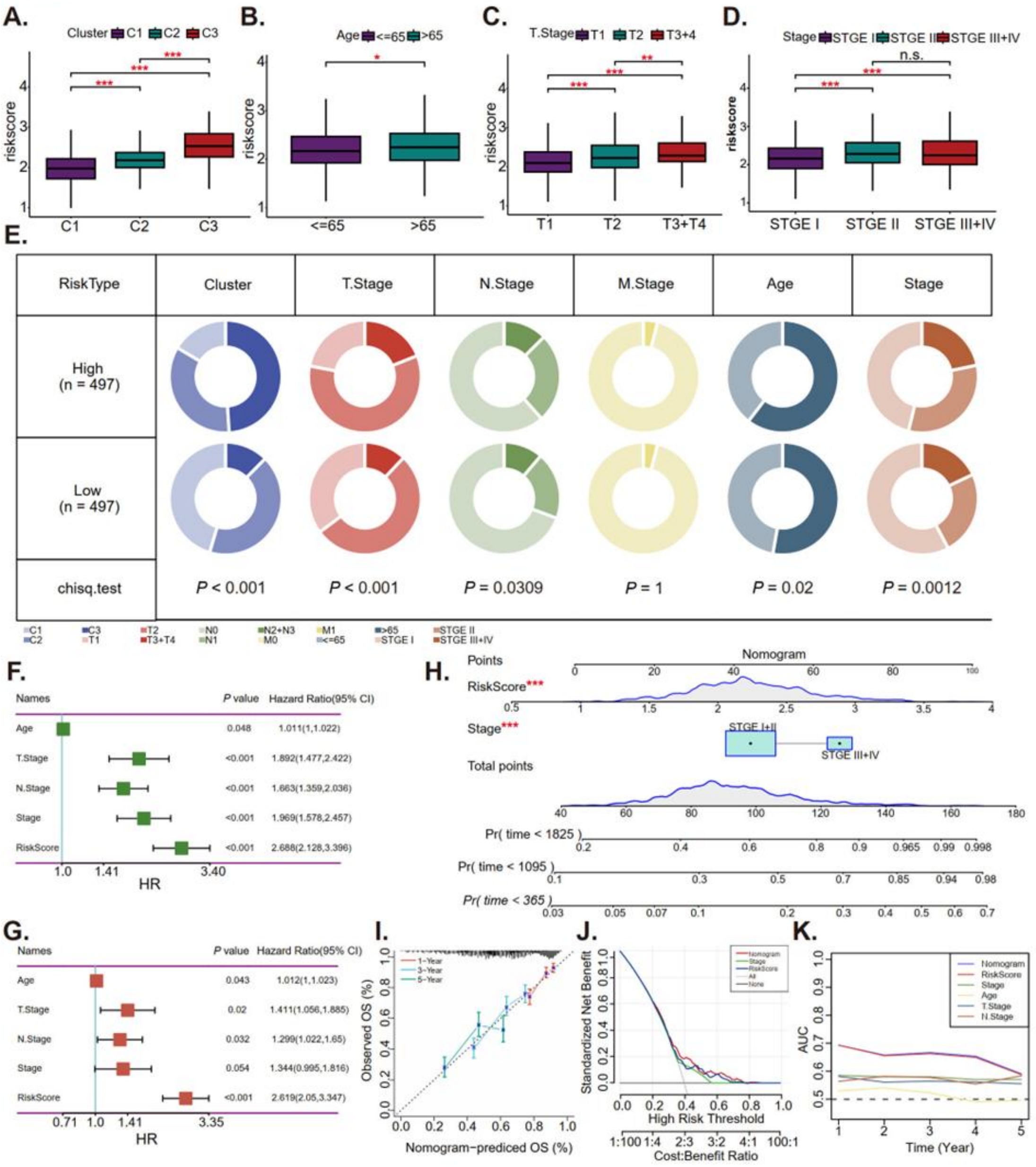

Fig.S2 Clinical characteristics and independent prognostic value of the model. Differences in risk score in different clinical subgroups in the TCGA-NSCLC cohort (**A-D**), including molecular subtypes (**A**), age (**B**), T stage (**C**), and AJCC stage (**D**). Pie charts showing the cardinality test for clinicopathological factors and LRrisk subgroups for each group of the LRriskscore (**E**). Independent prognostic value of the risk model (**F-K**). Univariate Cox (**F**) and multivariate cox (**G**) analysis to assess the independence of the model. Nomogram survival prediction of NSCLC patientswith risk score (**H**). Calibration plot of the nomogram (**I**). DCA was performed to present the net benefit of risk scorecompared to clinical parameters (**J**). The difference of predictive ability was shown in time-dependent ROC curve(**K**).**P*< 0.05; ***P*< 0.01; ****P*< 0.001; "n.s." stands for *P*> 0.05.

Fig.S3

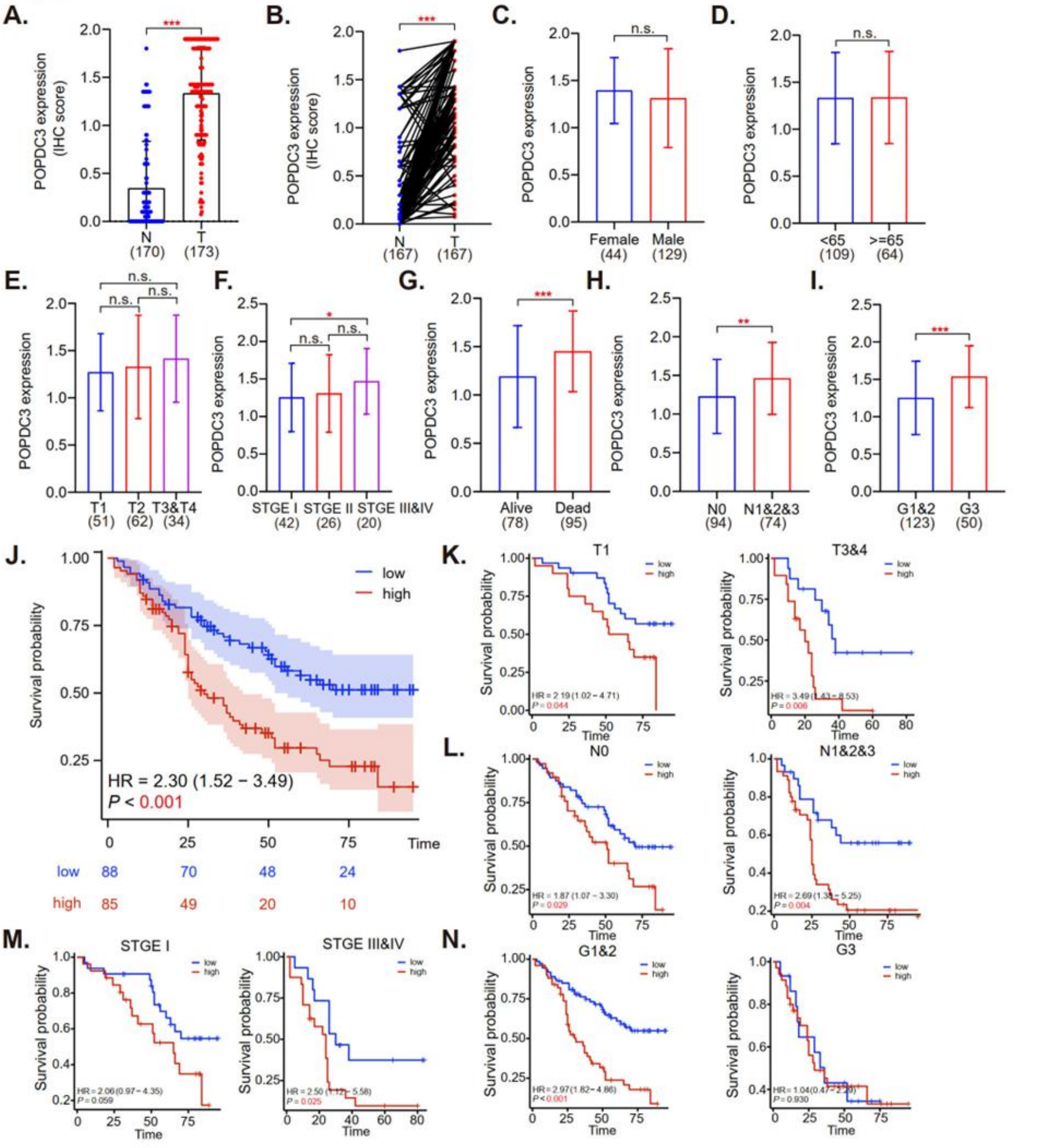

Fig.S3 POPDC3 expression with NSCLC clinical features. The interrelation of the POPDC3 IHC Score with clinicopathological characteristics, including unpaired tissues(**A**), paired tissues (**B**), gender (**C**), age (**D**), T stage (**E**), clinical stage (**F**), OS event (**G**), N stage (**H**), and pathological grade(**I**). The OS of NSCLCs between high expression and low expression of POPDC3 (**J**). Subgroup survival analysis in NSCLC patients. T stage (**K**). N stage (**L**). AJCC stage (**M**) and pathological grade (**N**). **P*< 0.05; ***P*< 0.01; ****P*< 0.001; "n.s." stands for *P*> 0.05.

Fig.S4

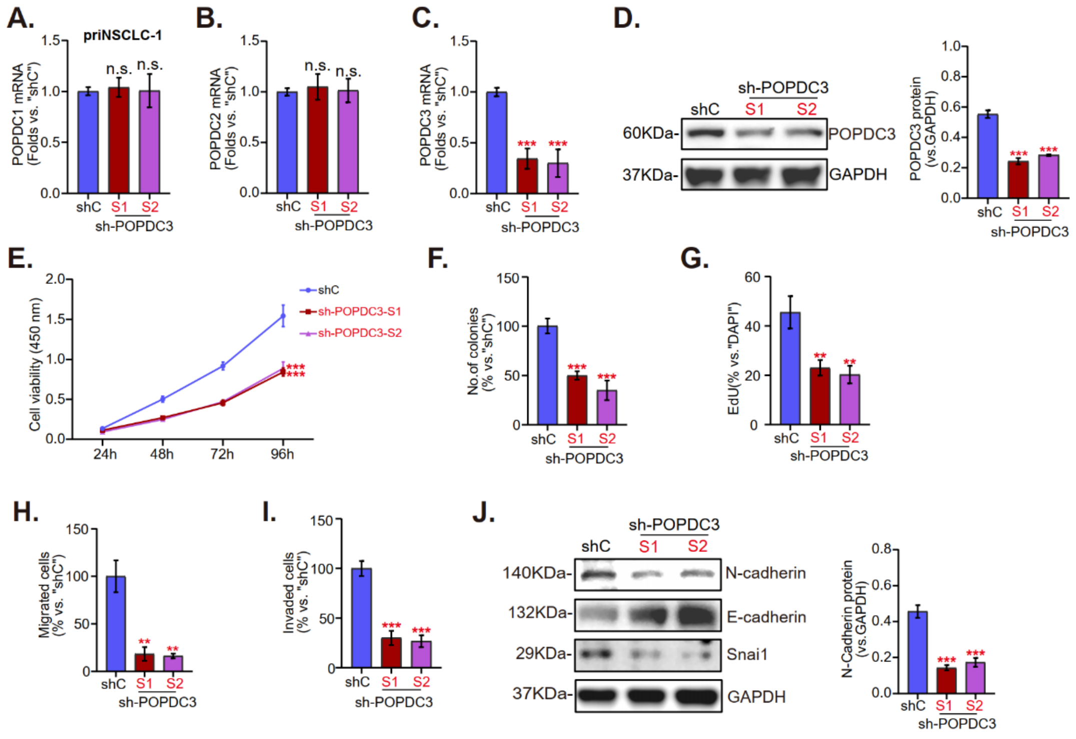

Fig.S4 POPDC3 silencing inhibits primaryNSCLC cells proliferation and invasion. Puromycin-selected priNSCLC-1 primary human NSCLC cells,with the applied POPDC3 shRNA ("sh-POPDC3-S1/S2", containing two different sequences) or the scramble control shRNA ("shc"), were cultured, and expression of listed genes and proteins was shown (**A-D**); Cells were further cultivated for indicated time periods, cell viability (**E**), colony formation (**F**) and EdU incorporation (**G**), in vitro cell migration (**H**) and invasion (**I**) were tested using the described methods, with results quantified. The expression of listed proteins was analyzed using Western blotting (**J**). "Pare" stands for the parental control cells. Error bars stand for mean ± standard deviation(SD, n = 3).Statistical significance is denoted as **P*< 0.05; ***P*< 0.01; ****P*< 0.001 versus "shc" cells, while "n.s." stands for *P*> 0.05.

Fig S5.

Fig6

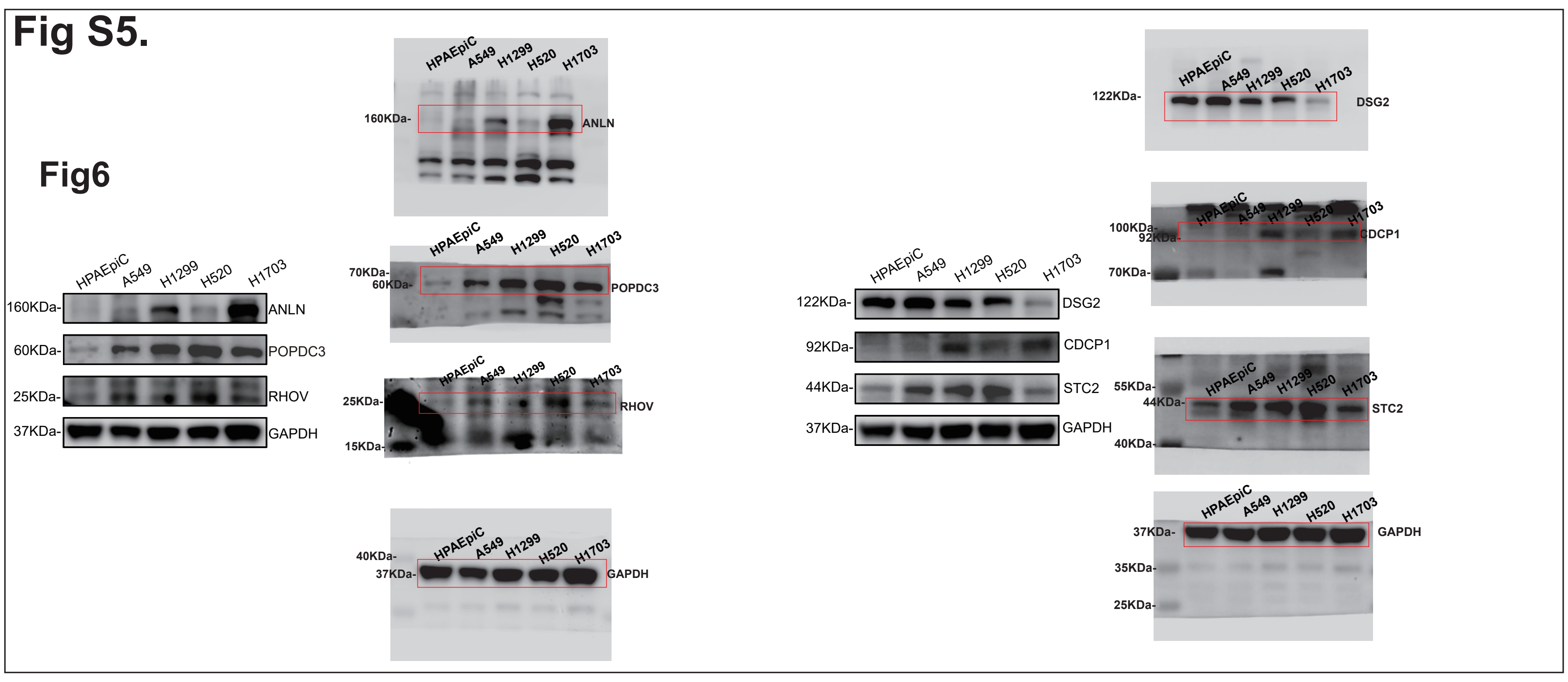

Fig8

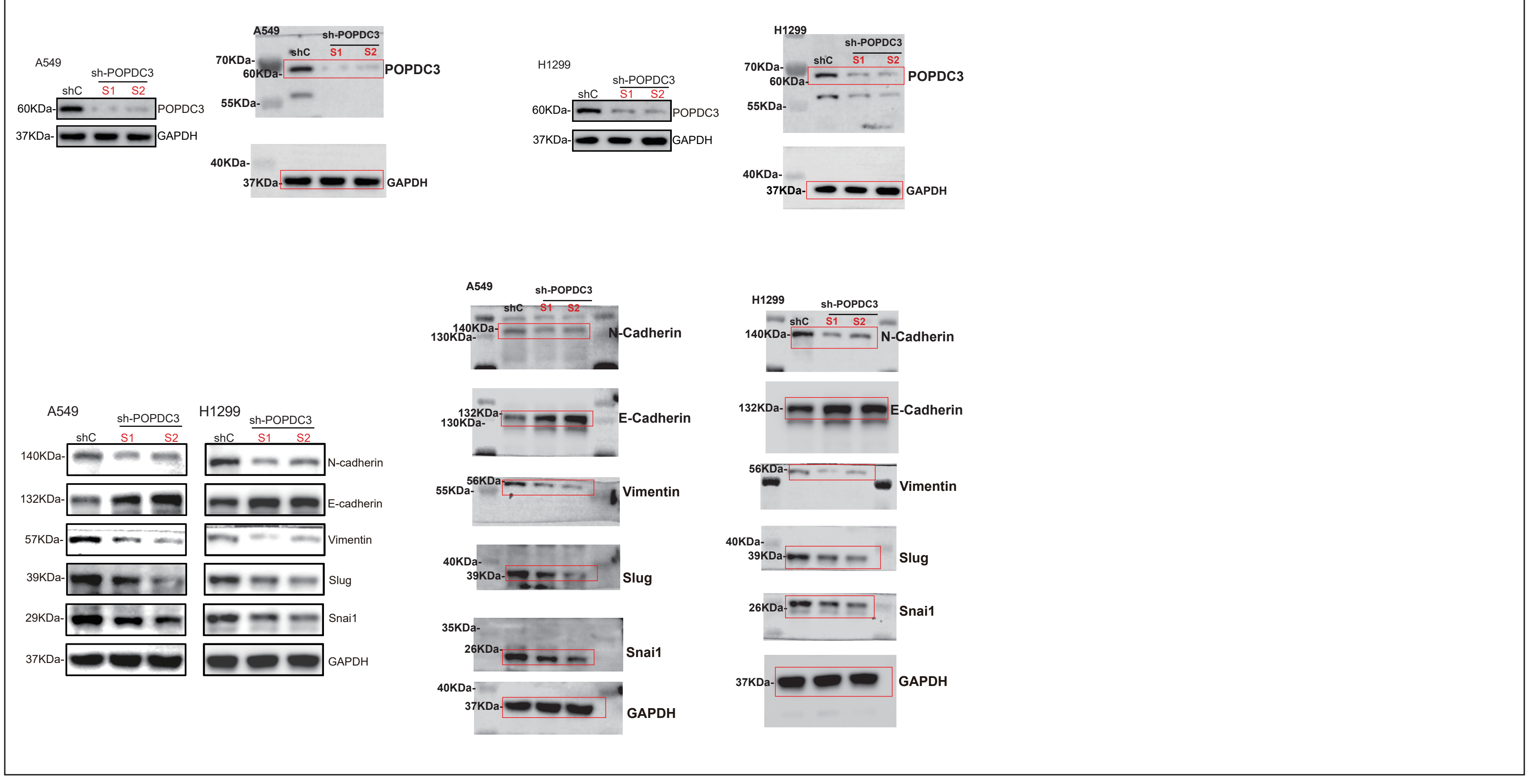

Fig9

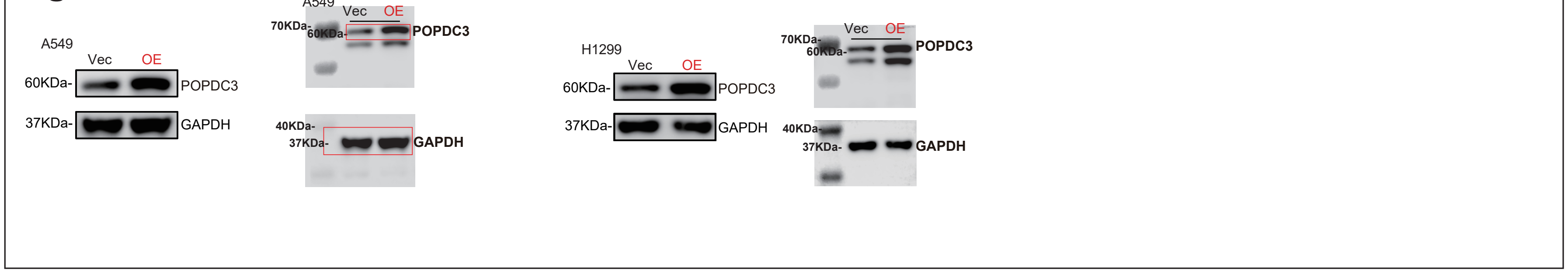

Fig10

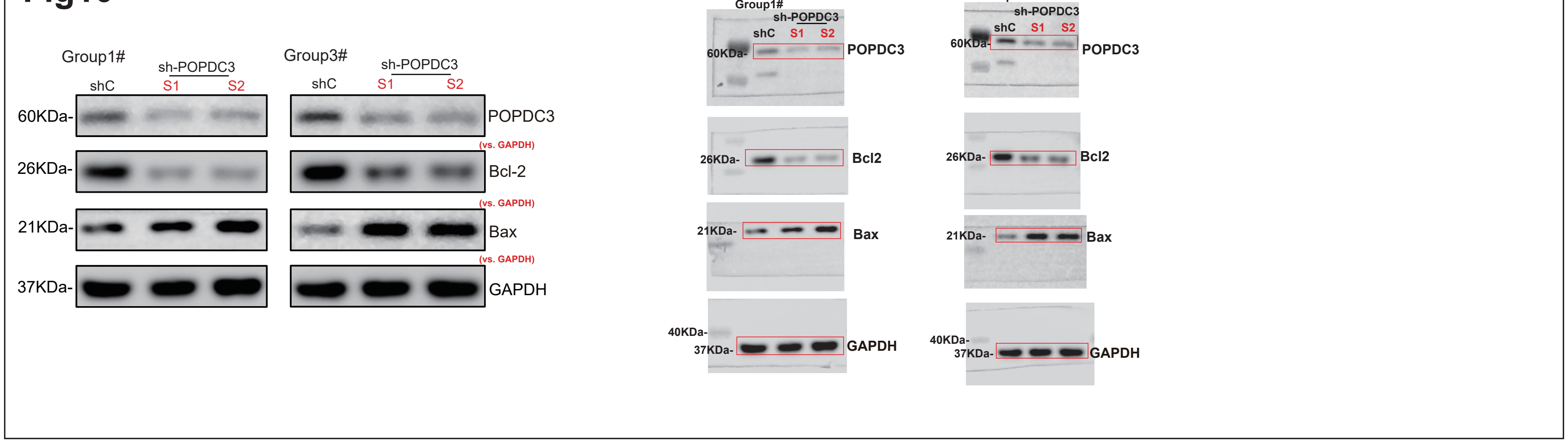

Fig11

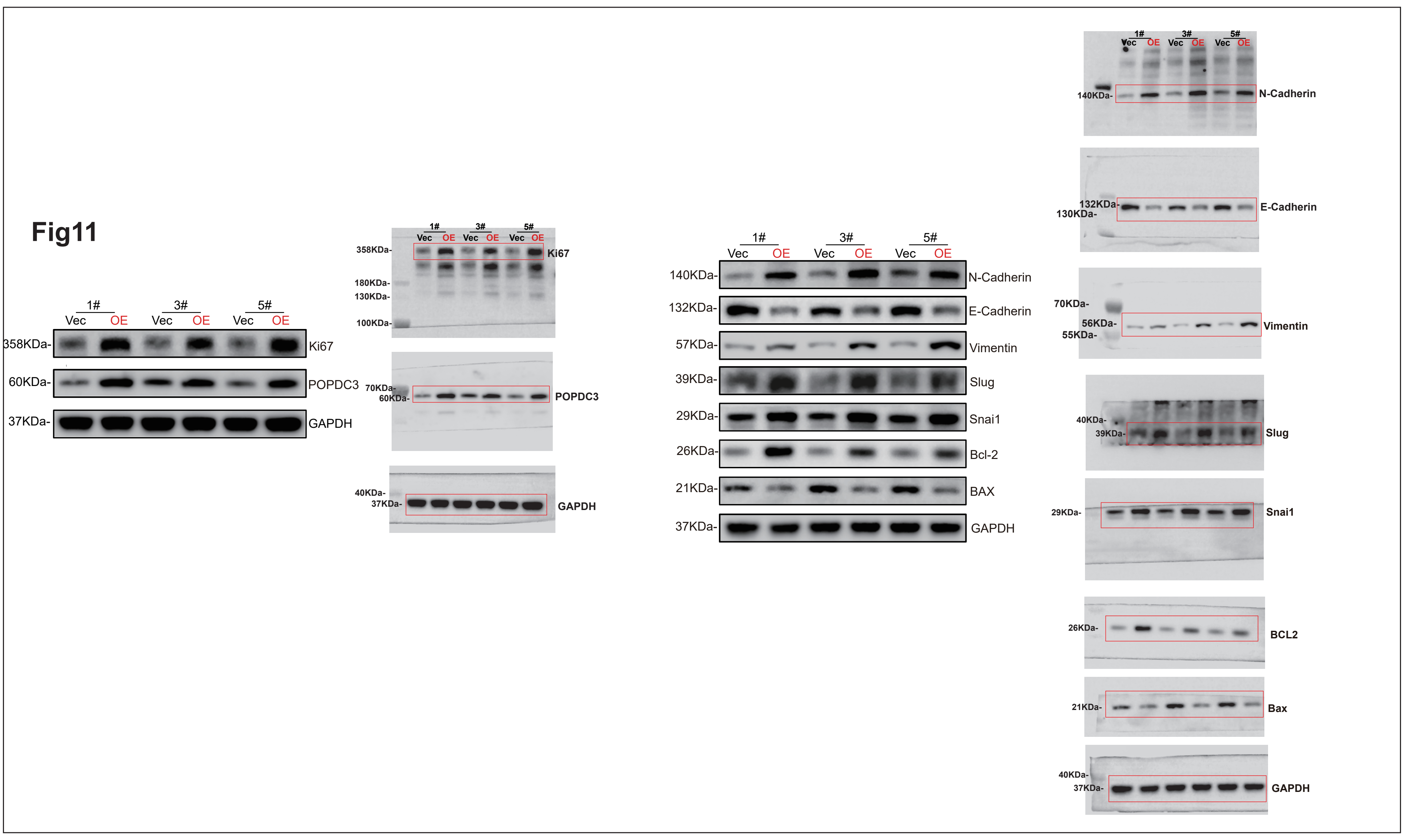

FigS4

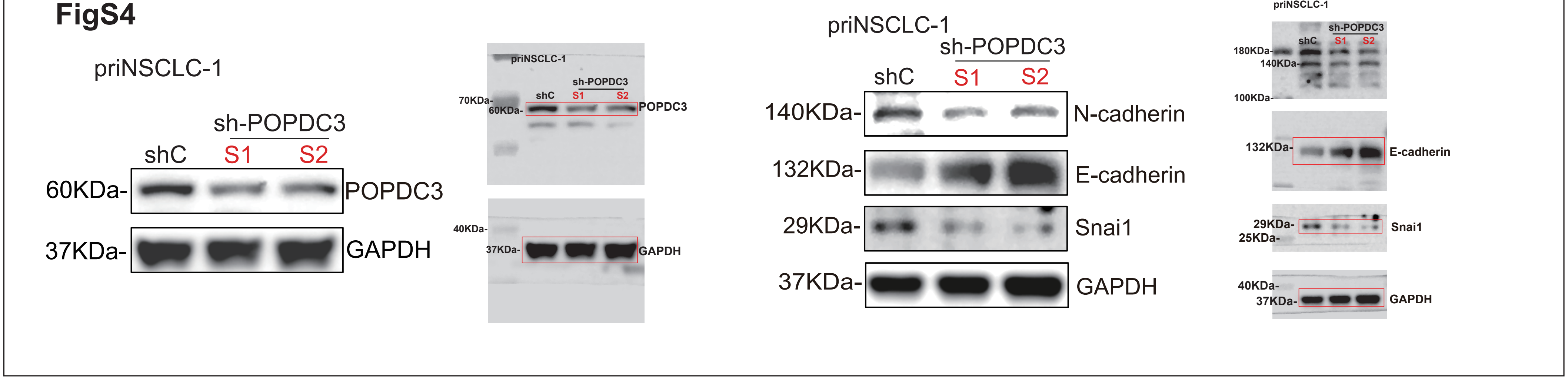

Fig S5: The uncropped blotting images of the study.
The uncropped Western blotting images corresponding to the cropped blots shown in each Figure are presented.
